# Potential Shared Immunometabolic Signatures Between Familial Hypercholesterolemia and Major Depressive Disorder: Integrative Transcriptomic Analysis, Mendelian Randomization, and Clinical Validation

**DOI:** 10.3390/ijms27177532

**Published:** 2026-08-22

**Authors:** Chenxi Liu, Yuting Li, Xiang Cao, Zixuan Ye, Quanzhou Shi, Feng Chen, Zihao Li, Jiamei Guo, Tian Qiu

**Affiliations:** Department of Psychiatry, The First Affiliated Hospital of Chongqing Medical University, Chongqing 400016, China; 1446596069@163.com (C.L.); dragonliyt@163.com (Y.L.);

**Keywords:** familial hypercholesterolemia, major depressive disorder, machine learning, Mendelian randomization, immune infiltration

## Abstract

Familial hypercholesterolemia (FH) has been associated with an increased risk of major depressive disorder (MDD), but their shared molecular signatures remain unclear. This study aimed to identify candidate genes associated with FH-MDD co-occurrence through integrative transcriptomic analysis, machine learning, summary-data-based Mendelian randomization (SMR), and clinical validation. Transcriptomic datasets from the Gene Expression Omnibus were analyzed, including GSE6054 and GSE13985 for FH and GSE98793 for MDD. Differential expression analysis and weighted gene co-expression network analysis were used to identify shared candidate genes. Candidate biomarkers were screened using least absolute shrinkage and selection operator regression and Random Forest algorithms, followed by the construction of an exploratory nomogram. SMR analysis was performed to assess genetically supported associations between candidate-gene expression and MDD risk. Selected genes were validated by PBMC RT-qPCR in an independent four-group clinical cohort comprising healthy controls and participants with FH, MDD, or co-occurring FH and MDD. Flow cytometry was subsequently used to characterize the peripheral CD4+ T-cell profile. A total of 54 shared candidate genes were identified. CD4, MRPS21, and CRTC2 were selected by machine learning and incorporated into the nomogram, which showed good discrimination in the training set and moderate performance in external validation. Immune infiltration and enrichment analyses highlighted monocyte alterations, reduced T-cell-related signals, and enrichment of T-cell receptor and TNF-mediated pathways. SMR analysis indicated that genetically predicted higher CD4 expression was inversely associated with MDD risk, whereas RT-qPCR showed higher CD4 expression in patients with FH-MDD. Flow-cytometric analysis showed that FH-MDD participants had a higher circulating CD3+CD4+ T-cell proportion, an increased effector-memory CD4+ T-cell proportion, and a higher proportion of HLA-DR+ CD4+ T cells than participants in the comparison groups. These findings suggest that CD4 may represent a promising immune-related candidate signature associated with the co-occurrence of FH and MDD, warranting further functional validation.

## 1. Introduction

Major depressive disorder (MDD) is a highly prevalent psychiatric disorder characterized by persistent low mood, anhedonia, behavioral changes, and a range of somatic symptoms [1]. The World Health Organization estimates that depression affects approximately 3.8% of the global population, corresponding to nearly 280 million people worldwide [2]. The pathophysiology of MDD is complex and may involve monoaminergic neurotransmitter dysregulation, alterations in the brain–gut axis, dysfunction of the hypothalamic–pituitary–adrenal (HPA) axis, impaired neurotrophic signaling, and neuroinflammatory processes [3]. However, treatments developed on the basis of these mechanisms remain insufficiently effective in a substantial proportion of patients, indicating persistent gaps in our understanding of the core biological mechanisms underlying depression [4].

Familial hypercholesterolemia (FH) is a common inherited lipid disorder characterized by lifelong elevation of low-density lipoprotein cholesterol (LDL-C), most commonly due to pathogenic variants affecting the LDL receptor pathway [5]. Approximately 450,000 children worldwide are born with FH each year, leading to sustained LDL-C exposure from early life [6]. In addition to its well-established cardiovascular consequences, accumulating evidence suggests that FH may also be associated with psychological and neuropsychiatric manifestations. Depressive symptoms in patients with FH have been reported to correlate with LDL-C levels [7], and clinical studies have suggested a higher prevalence of anxiety and depressive symptoms among individuals with FH [8]. Experimental studies have similarly demonstrated depression-like behavior in LDL receptor-deficient mice, accompanied by abnormalities in hippocampal and cortical monoamine metabolism [9]. Impaired adult hippocampal neurogenesis has also been observed in FH mouse models, further supporting a potential relationship between LDL receptor-related cholesterol metabolism and emotional regulation [10]. Several biological mechanisms may link FH-related lipid dysregulation to depressive pathophysiology. Unlike acquired dyslipidemia, FH is characterized by lifelong, genetically determined LDL-C elevation, which may exert sustained effects on vascular, immune, and neurobiological processes. Cholesterol is essential for neuronal membrane integrity, synaptic stability, and neurotransmission, and its dysregulation may influence monoaminergic signaling, hippocampal neurogenesis, and neuroimmune communication. Hypercholesterolemia has also been associated with overactivation of the hypothalamic–pituitary–adrenal axis, a well-established component of depressive pathophysiology [11]. In addition, chronic lipid overload may promote systemic inflammation and immune-cell dysfunction, providing a plausible link between metabolic abnormalities and altered neuroimmune regulation.

Although previous studies have reported associations between FH and MDD, several important questions remain unresolved. First, most available evidence has been derived from relatively small clinical cohorts or experimental models, and few studies have integrated transcriptomic data to identify molecular features shared by the two disorders. Consequently, the candidate genes and biological pathways associated with the clinical co-occurrence of FH and MDD remain poorly defined. Second, most studies remain at the correlational level, making it difficult to determine whether FH is an initiating factor for MDD [12]. Recent advances in Mendelian randomization (MR) and quantitative trait locus (QTL) analyses provide genetically informed evidence that may help prioritize disease-associated genes [13].

To address these gaps, we integrated transcriptomic analysis, weighted gene co-expression network analysis, machine learning, immune-cell deconvolution, and summary-data-based Mendelian randomization to identify candidate molecular signatures shared by FH and MDD. Selected genes were evaluated in an independent four-group clinical cohort comprising healthy controls and participants with FH, MDD, or co-occurring FH and MDD. PBMC-based RT-qPCR was used to validate candidate-gene expression, and flow cytometry was performed to characterize the peripheral CD4+ T-cell profile. This study aimed to identify exploratory immune-related candidates associated with FH-MDD co-occurrence rather than to establish a definitive causal mechanism.

## 2. Results

### 2.1. Demographic Characteristics of the Four-Group Clinical Cohort

A total of 120 participants were included in the clinical validation cohort, with 30 participants in each of the CON, FH, MDD, and FH-MDD groups. There were no significant differences among the four groups in age, sex distribution, BMI, white blood cell count, C-reactive protein, urea, creatinine, or uric acid levels (all *p* > 0.05). HAMD-24 scores were significantly higher in the MDD and FH-MDD groups than in the CON and FH groups (*p* < 0.001), whereas LDL-C levels were significantly higher in the FH and FH-MDD groups than in the CON and MDD groups (*p* < 0.001) (Table 1). Thus, the four groups were generally comparable in demographic characteristics and other measured clinical variables, apart from the expected differences in HAMD-24 scores and LDL-C levels.

### 2.2. Identification of DEGs in FH and MDD Datasets

**Figure 1:**
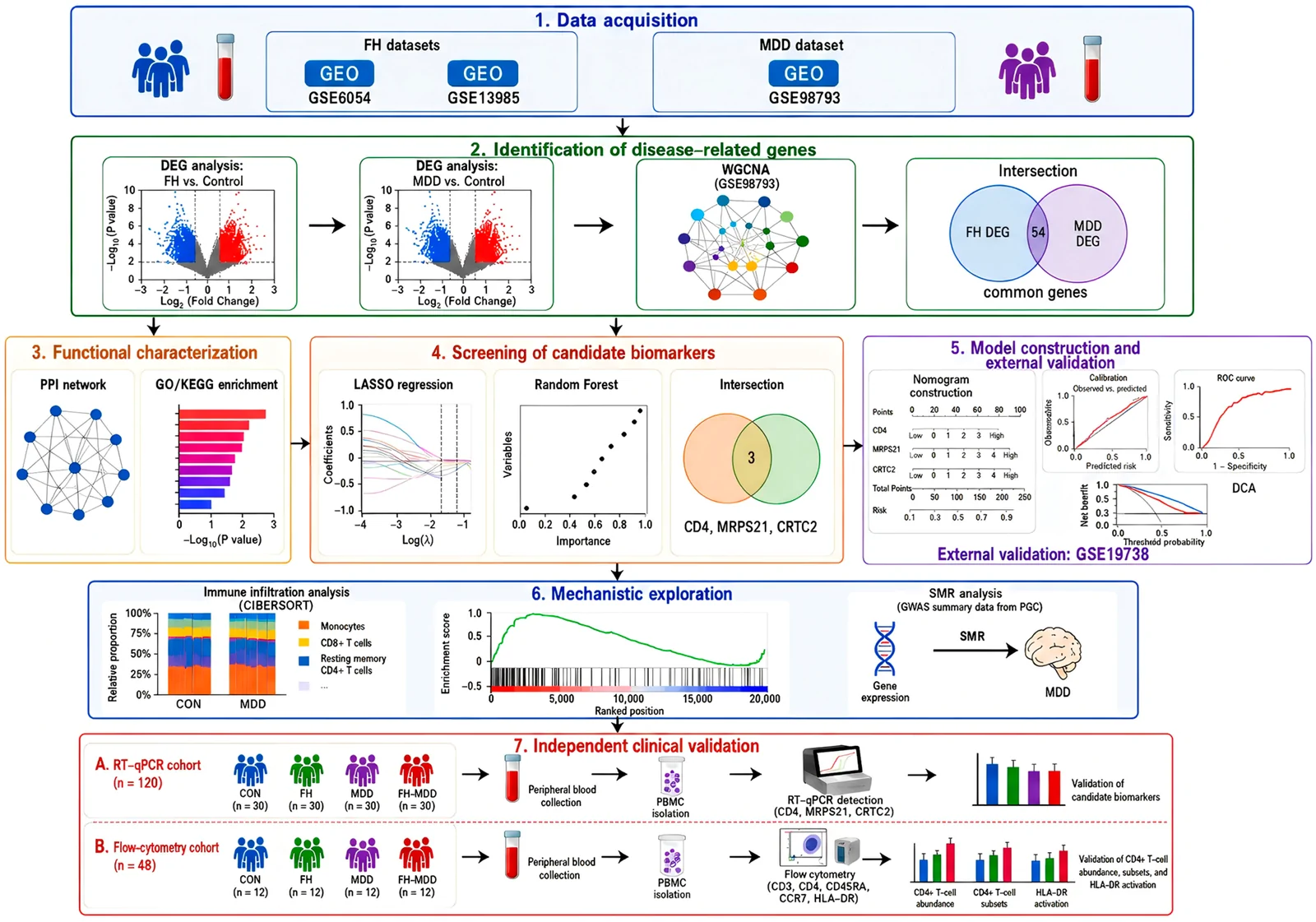
Flow chart of this study. Created with the assistance of ChatGPT, OpenAI, GPT-5.5 Thinking, accessed on 15 May 2026, and revised and verified by the authors.

**Figure 2:**
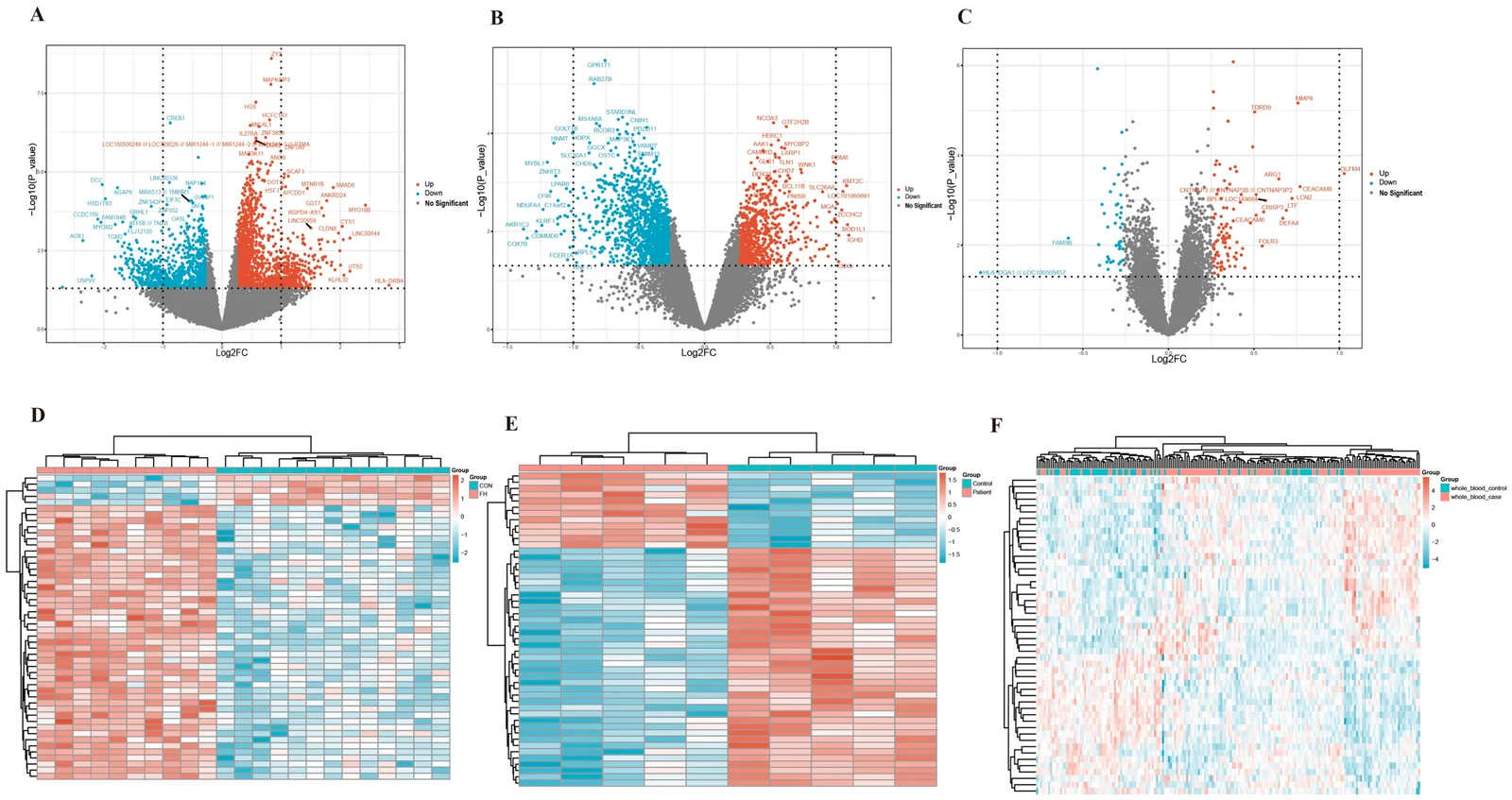
Integration and differential expression analysis of FH and MDD datasets. (**A**) Volcano plot of DEGs in the GSE6054 dataset; (**B**) volcano plot of DEGs in the GSE13985 dataset; (**C**) volcano plot of DEGs in the GSE98793 dataset; (**D**) heatmap of DEGs in the GSE6054 dataset; (**E**) heatmap of DEGs in the GSE13985 dataset; (**F**) heatmap of DEGs in the GSE98793 dataset.

The analytical workflow for this study is illustrated in Figure 1. Peripheral blood expression data were obtained from GSE6054 and GSE13985 for FH and from GSE98793 for MDD. After data preprocessing and normalization, DEGs were identified using the limma package (R software (R 4.4.2)) with thresholds of *p* < 0.05 and |log2FC| > 0.585. A total of 2180 DEGs, including 1300 upregulated and 880 downregulated genes, were identified in GSE6054 (Figure 2A,D). The GSE13985 dataset revealed 1787 DEGs, comprising 634 upregulated genes and 1153 downregulated genes (Figure 2B,E). The intersection of the two FH datasets identified 195 common DEGs. In GSE98793, 142 DEGs were identified, comprising 100 upregulated and 42 downregulated genes (Figure 2C,F). Volcano plots and heatmaps were used to visualize all DEGs. As an additional multiple-testing assessment, FDR-adjusted *p* values were calculated for all genes and reported in the supplementary DEG tables (Supplementary Table S1). With the stringent threshold of FDR-adjusted *p* < 0.05, 200 DEGs (168 upregulated and 32 downregulated), no DEGs, and 6 DEGs (5 upregulated and 1 downregulated) were identified from GSE6054, GSE13985 and GSE98793, respectively. The *p* value threshold was retained only to enable exploratory integrative screening. All genes selected at the *p* value threshold, as well as the candidate genes derived from subsequent intersections and machine-learning analyses, should be interpreted as hypothesis-generating candidates rather than FDR-confirmed disease-associated genes.

### 2.3. Construction of Weighted Gene Co-Expression Networks and Identification of Key Modules in MDD

**Figure 3:**
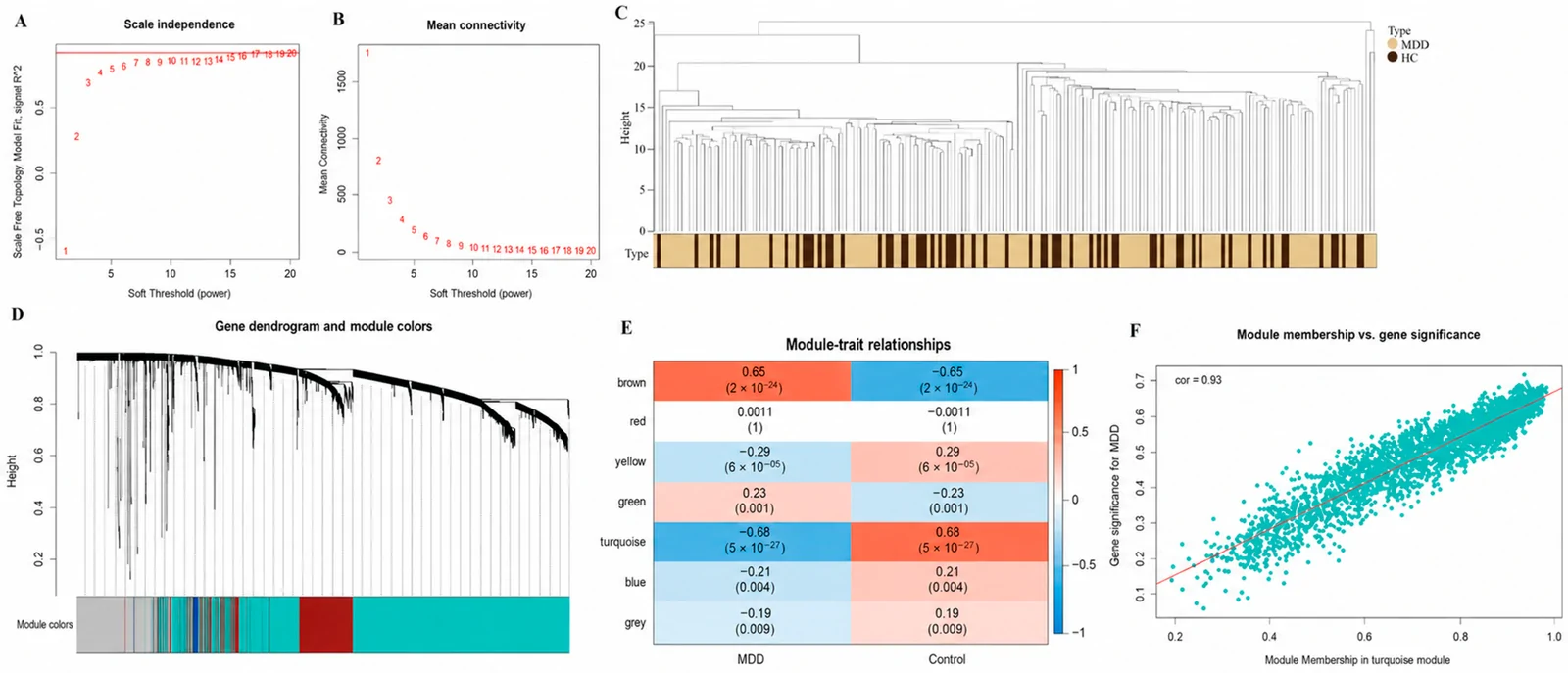
Identification of key module genes in the MDD dataset using WGCNA. (**A**,**B**) Determination of optimal β value using a scale-free topological model; β = 10 selected as soft threshold based on average connectivity and scale independence. (**C**,**D**) Gene dendrograms. (**E**) Heatmap revealing relationships between module signature genes and MDD status. The turquoise and brown modules showed the highest correlation coefficients with MDD and were identified as key modules for MDD. (**F**) Correlation plot between turquoise module membership and gene significance within the turquoise module.

WGCNA was performed to identify gene modules associated with MDD. A soft-thresholding power of β = 10 was selected to approximate scale-free topology while maintaining adequate network connectivity (Figure 3A–D). Seven co-expression modules were identified (Figure 3E). Correlation analysis revealed that the turquoise and brown modules showed extremely high correlations with MDD (brown module, r = 0.65, *p* = 2 × 10^−24^; turquoise module, r = 0.68, *p* =2 × 10^−27^) (Figure 3F). These modules contained 2580 and 619 genes, respectively. Intersecting the 3199 module genes with the 195 FH-related DEGs yielded 54 candidate genes associated with shared FH- and MDD-related transcriptional features.

### 2.4. Protein–Protein Interaction Network and Functional Enrichment of Candidate Shared Genes in FH and MDD

**Figure 4:**
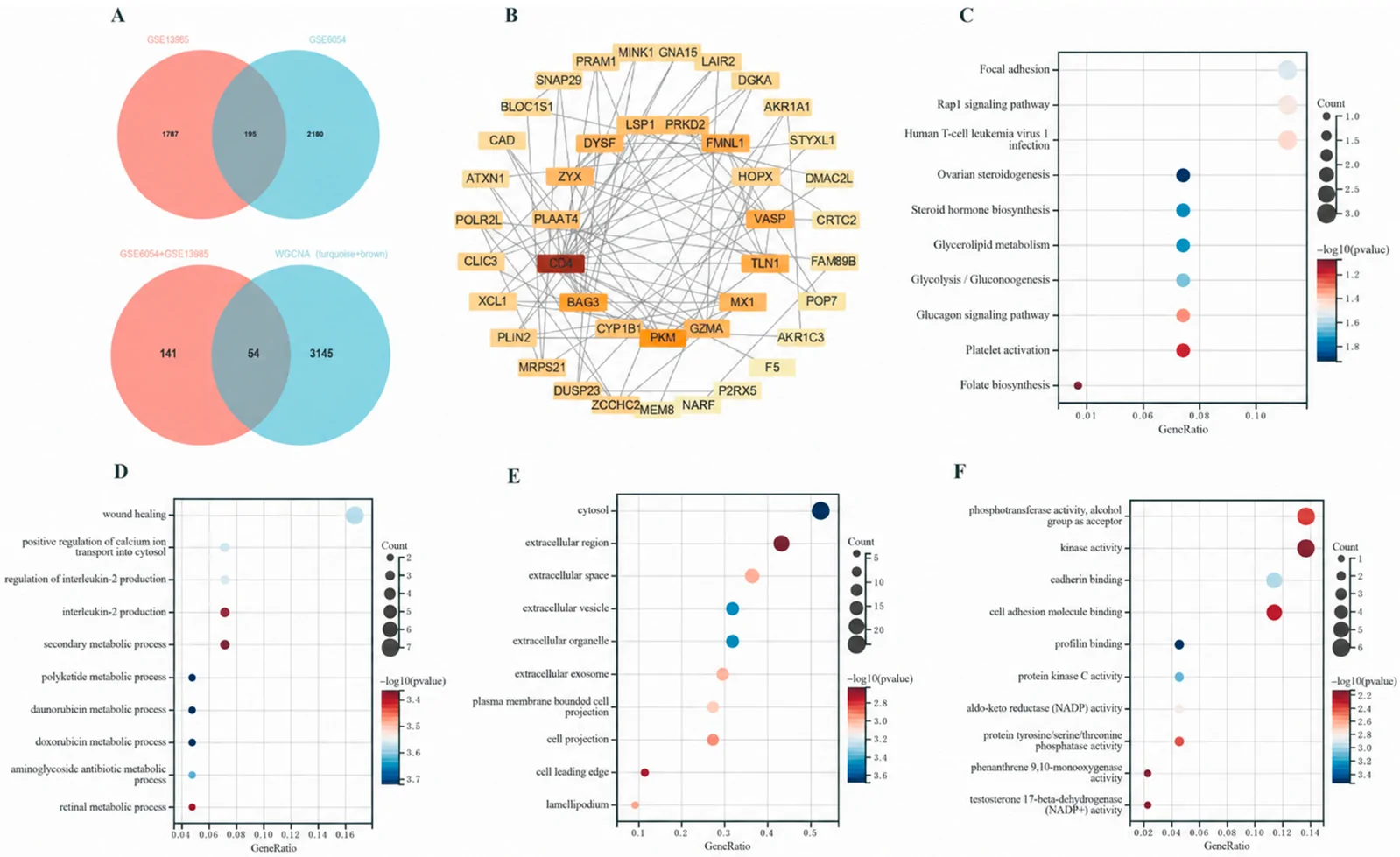
Protein–protein interaction (PPI) analysis and related enrichment analysis of FH-MDD key genes. (**A**) Venn diagram showing the intersection of DEGs from FH datasets GSE6054 and GSE13985, yielding 195 intersecting genes. Further intersection with key MDD module genes yielded 54 overlapping genes; (**B**) PPI network of FH-MDD key genes based on the highest-scoring module genes from MCODE analysis in Cytoscape (v3.7.1); (**C**) bubble chart of KEGG enrichment analysis results; (**D**–**F**) bubble chart of GO enrichment analysis results.

Protein–protein interaction analysis of the 54 candidate shared genes was performed using the STRING database (Figure 4A). A PPI network comprising 42 genes was constructed for subsequent functional annotation (Figure 4B). GO and KEGG enrichment results were evaluated using FDR-adjusted *p* values, with FDR-adjusted *p* < 0.05 considered statistically significant. The candidate genes were primarily enriched in processes related to cell adhesion and migration, immune regulation, metabolic reprogramming, and intercellular communication. KEGG analysis identified enrichment in focal adhesion, Rap1 signaling, human T-cell leukemia virus type 1 infection, glycolysis, glyceride metabolism, and folate synthesis (Figure 4C). GO biological process analysis indicated enrichment in wound healing, regulation of interleukin-2 production, and drug metabolism (Figure 4D). Cellular component terms included the cytoplasm, extracellular vesicles, and migration-associated structures such as lamellipodia (Figure 4E). Enriched molecular functions included kinase activity, cell adhesion molecule binding, and protein kinase C activity (Figure 4F).

### 2.5. Machine Learning-Based Identification of Candidate Feature Genes

**Figure 5:**
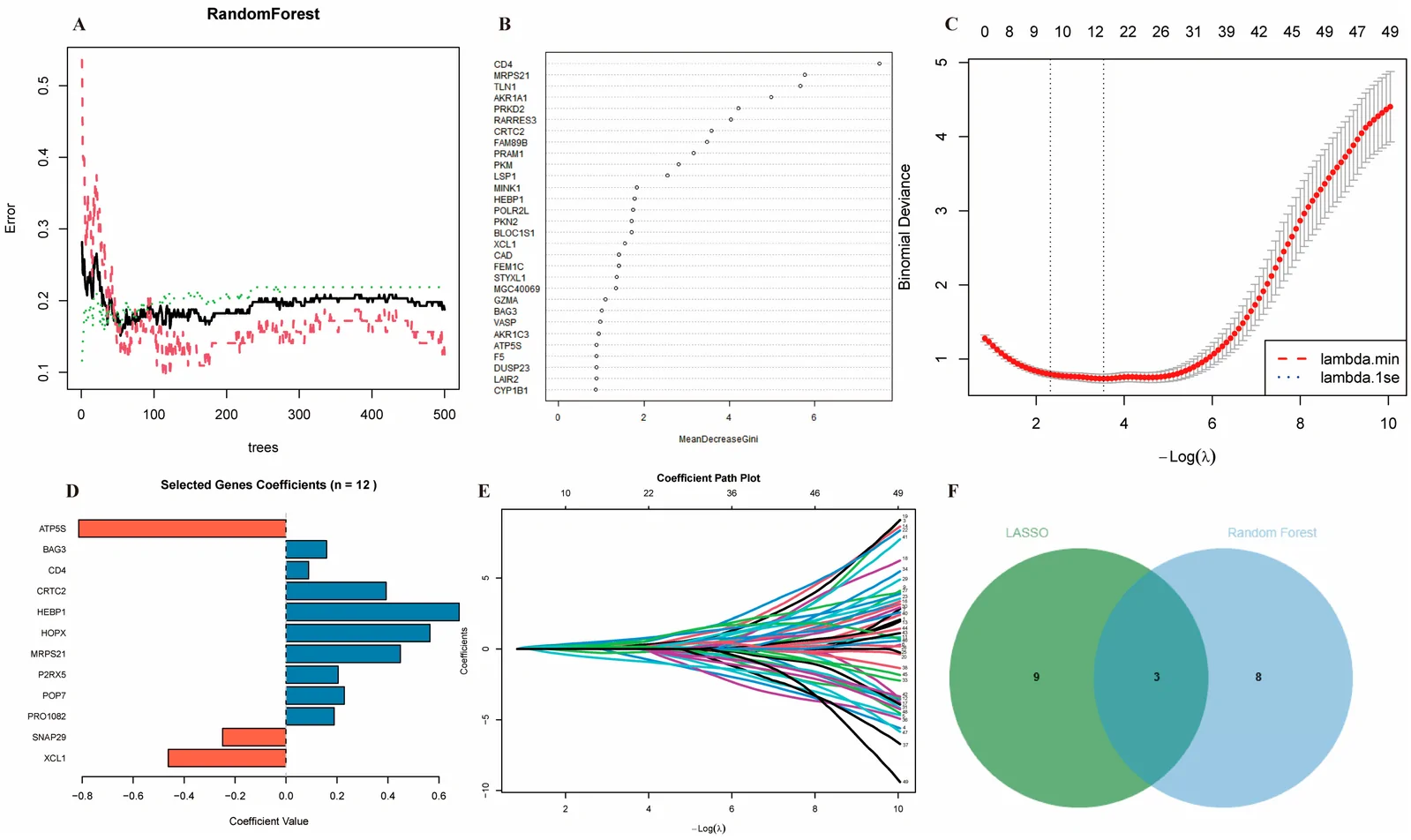
Identification of candidate feature genes using LASSO regression and Random Forest analyses. (**A**) Random Forest error rates as a function of the number of trees. The black line represents the overall out-of-bag (OOB) error rate, while the red and green lines represent the class-specific error rates for healthy controls and MDD patients, respectively. (**B**) MeanDecreaseGini plot of the 54 genes in MDD generated using the Random Forest (RF) algorithm. (**C**–**E**) Selection of candidate feature genes using LASSO logistic regression. (**F**) Intersection of candidate genes selected by LASSO and RF algorithms, yielding three candidate feature genes.

The expression profiles of the 54 candidate genes were analyzed using Random Forest and LASSO regression to prioritize candidate feature genes. Random Forest analysis with 500 trees and a MeanDecreaseGini threshold of >2 identified 11 candidate genes (Figure 5A,B). LASSO regression with λ = 0.03 selected 12 candidate genes (Figure 5C–E). The intersection of the two approaches yielded three candidate feature genes associated with overlapping FH- and MDD-related transcriptional signatures (Figure 5F).

**Figure 6:**
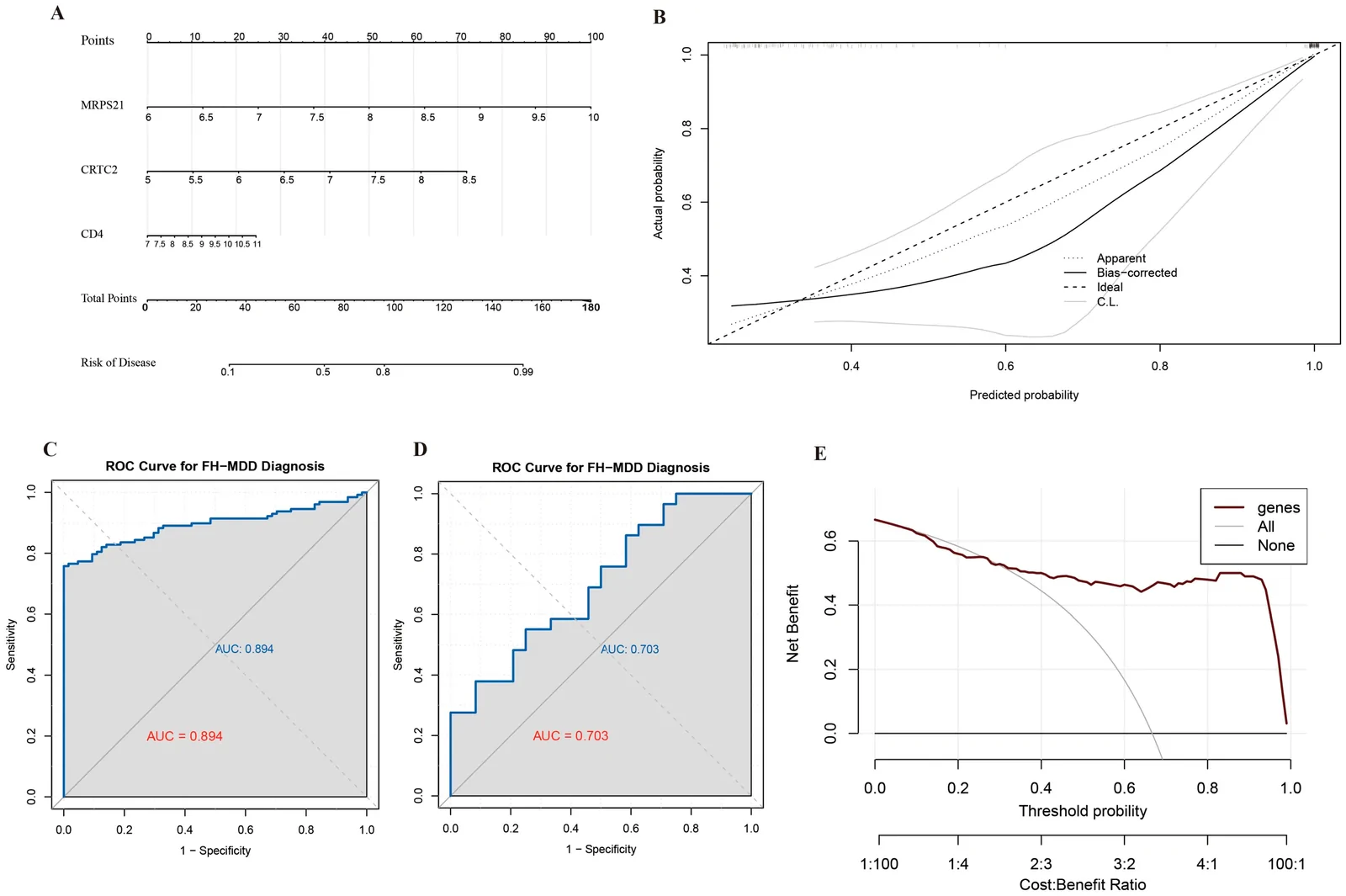
Development and evaluation of the exploratory nomogram model. (**A**) Nomogram constructed based on candidate biomarkers. (**B**) Calibration curve of the exploratory nomogram model. The dashed line labeled “Ideal” represents the standard curve, indicating perfect prediction by an ideal model. The dashed line labeled ‘Apparent’ denotes the uncalibrated prediction curve, while the solid line labeled “Bias-Corrected” represents the calibrated prediction curve. (**C**) Receiver operating characteristic (ROC) curve showing the discriminative performance of the nomogram in the training dataset of the GEO database. (**D**) ROC curve for the diagnostic performance of the nomogram model predicting MDD in the external dataset of the GEO database. (**E**) DCA of the nomogram model. The black line labeled “None” represents the net benefit assuming no patients have MDD. The gray line labeled ‘All’ represents the net benefit assuming all patients have MDD, while the red line labeled “Model” represents the net benefit assuming identification of FH-MDD based on the columnar graph model.

Logistic regression was subsequently used to construct an exploratory nomogram incorporating CD4, MRPS21, and CRTC2 (Figure 6A). Calibration analysis in the training cohort (n = 192) showed good agreement between predicted and observed probabilities, and the Hosmer–Lemeshow test showed no significant evidence of poor fit (*p* = 0.112; Figure 6B). Receiver operating characteristic (ROC) curve analysis (AUC = 0.894, 95% CI = 0.849–0.944) showed good discriminative performance of the exploratory model in the training dataset (Figure 6C). To further assess model stability, bootstrap resampling with 1000 iterations was performed in the training dataset, yielding a bootstrap-corrected AUC of 0.858 (95% CI: 0.804–0.912). External validation using the independent dataset GSE19738 yielded an AUC of 0.703 (95% CI: 0.648–0.759), indicating moderate external discrimination for MDD classification (Figure 6D). Decision curve analysis (DCA) was used to explore the potential clinical netbenefit of the nomogram (Figure 6E). As a sensitivity analysis, we evaluated the cross-dataset consistency of the three prioritized genes using inverse-variance-weighted meta-analysis of GSE6054 and GSE13985. CD4 showed concordant upregulation across both FH datasets and a significant pooled effect after Benjamini–Hochberg correction (pooled log2FC = 0.371, 95% CI: 0.266–0.476, adjusted *p* = 1.66 × 10^−10^). CRTC2 also showed a concordant and significant pooled effect, whereas MRPS21 showed discordant directions of effect, a non-significant pooled estimate, and substantial between-dataset heterogeneity. (Supplementary Tables S2 and S3 and Supplementary Figures S1–S3).

### 2.6. Immune Infiltration Analysis and Single-Gene Enrichment Analysis

**Figure 7:**
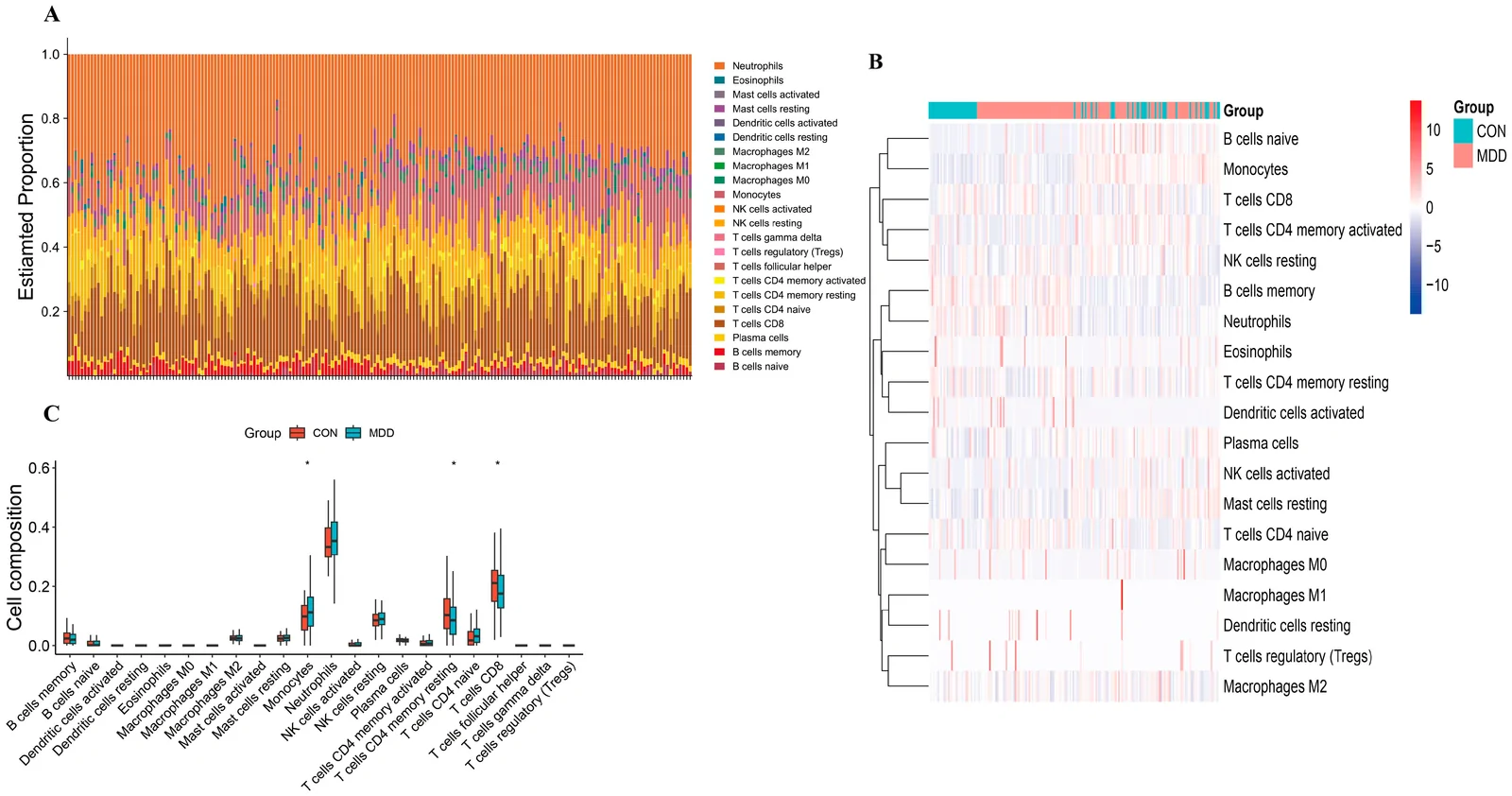
Immune cell infiltration analysis using CIBERSORT. Immune cell infiltration analysis of the GSE98793 dataset was performed using the CIBERSORT algorithm. (**A**) CIBERSORT analysis of different immune cell components; (**B**) distribution of feature scores for various immune cell types within clusters; (**C**) heatmap of immune cell type features and cluster analysis. * *p* value < 0.05.

CIBERSORT was used to estimate the relative proportions of 22 immune-cell types in GSE98793. Comparisons using the Wilcoxon rank-sum test indicated that MDD samples had a higher monocyte-related transcriptomic signal and lower CD8+ T-cell and resting memory CD4+ T-cell signals than control samples (Figure 7A–C).

**Figure 8:**
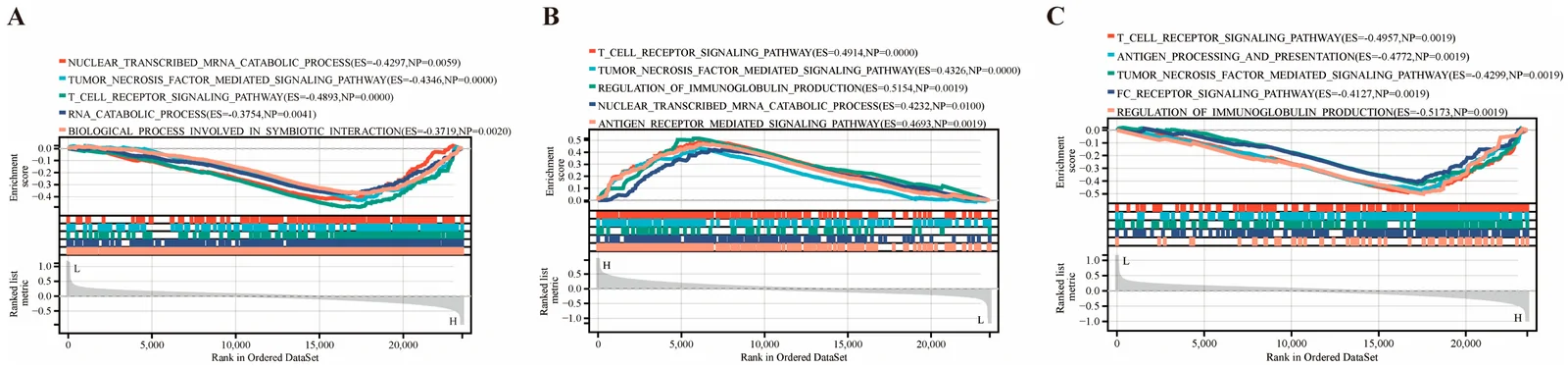
Single-gene enrichment analysis of candidate biomarkers. (**A**) Single-gene enrichment analysis of MRPS21; (**B**) single-gene enrichment analysis of CRTC2; (**C**) single-gene enrichment analysis of CD4.

Single-gene GSEA of the three candidate genes showed enrichment in biological processes related to T-cell receptor signaling, nuclear mRNA degradation, TNF-mediated signaling, antigen processing and presentation, and Fc receptor signaling (Figure 8A–C). Together with the immune-deconvolution results, these findings suggest that T-cell-related pathways may contribute to the shared immune-related transcriptional features of FH and MDD.

### 2.7. SMR Analysis of Candidate Genes

**Figure 9:**
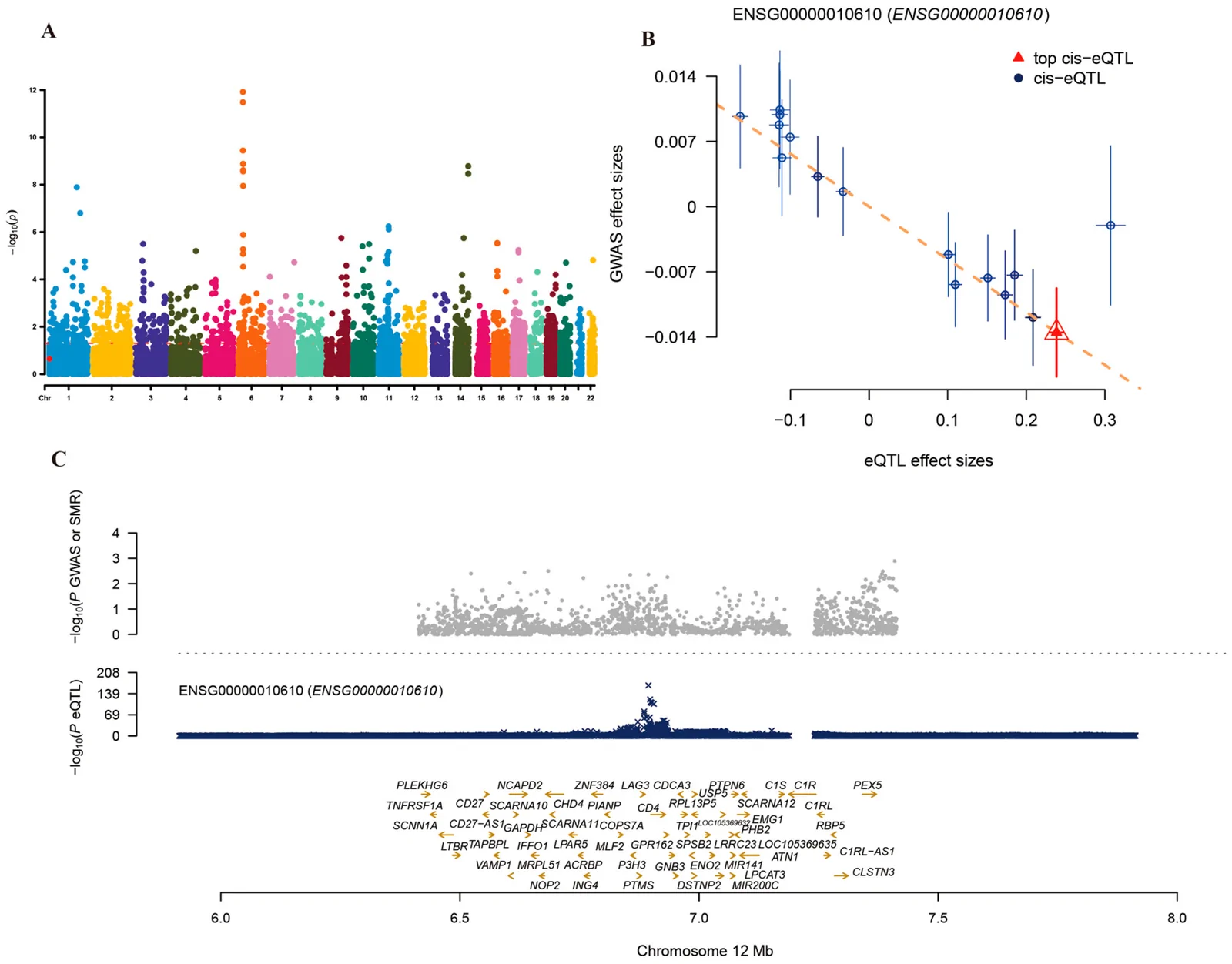
Mendelian randomization analysis of MDD and gene expression levels. (**A**) Manhattan plot of SMR results. (**B**,**C**) Summary of the relationship between MDD and CD4 expression levels in the Mendelian randomization study.

The Manhattan plot showed multiple significant chromosomal signals, with the strongest cluster located on chromosome 6. Additional independent peaks were observed on chromosomes 1, 3, 14, 15, 16, and 19 (Figure 9A).

SMR analysis was used to evaluate genetically supported associations between the expression of the three candidate genes and MDD risk. Genetically predicted CD4 expression was associated with lower MDD risk (*p* = 0.004, HEIDI score = 0.872), whereas no statistically significant SMR association was observed for MRPS21 (*p* = 0.056, HEIDI score = 0.167) or CRTC2 (*p* = 0.343, HEIDI score = 0.251). The HEIDI test did not indicate significant heterogeneity for the three candidate genes. The correlation diagram between CD4 and depression indicated that CD4 expression was inversely associated with MDD risk (Figure 9B,C).

### 2.8. Clinical Validation of Candidate Biomarkers in the Four-Group Cohort

**Figure 10:**
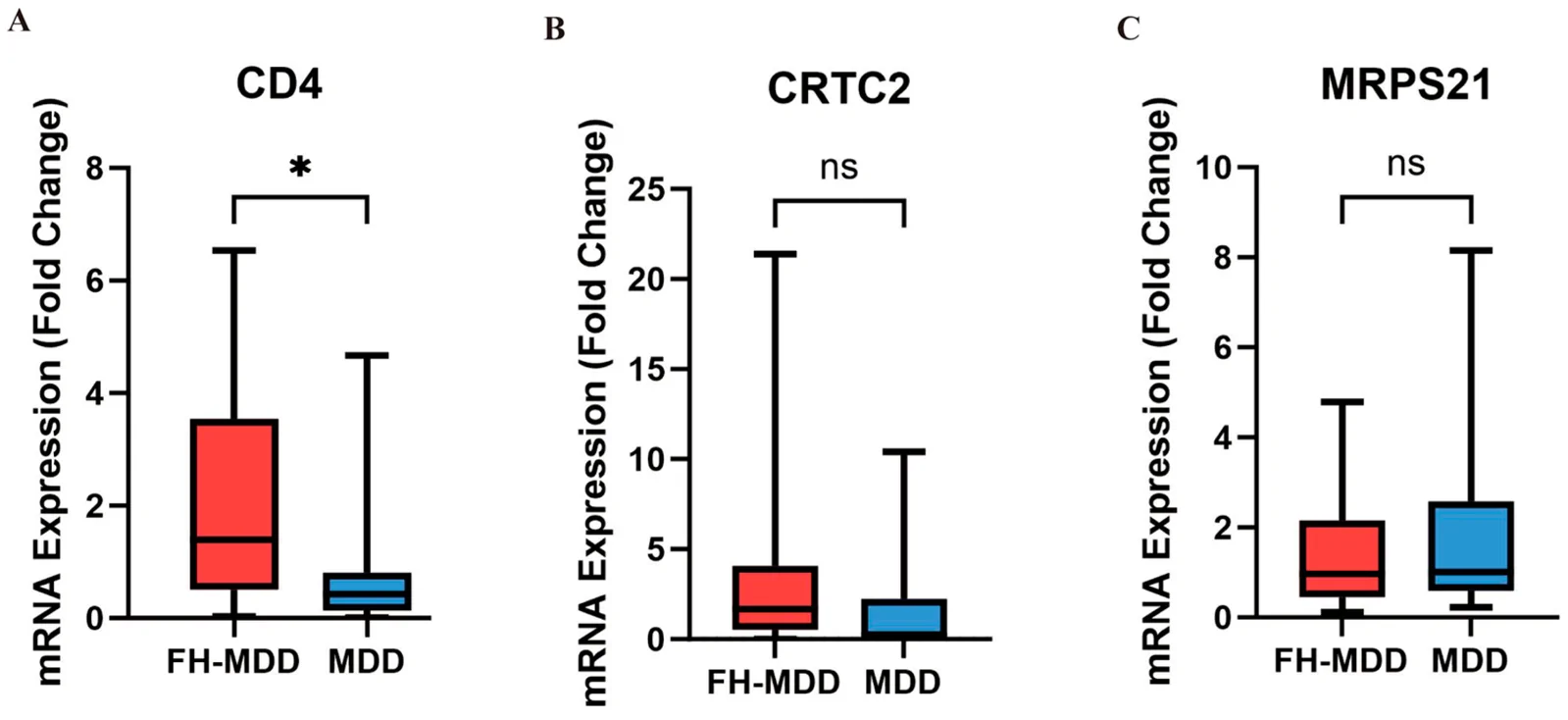
RT-qPCR validation of CD4, MRPS21, and CRTC2 expression in PBMCs. (**A**) Relative mRNA expression of CD4; (**B**) relative mRNA expression of MRPS21; (**C**) relative mRNA expression of CRTC2. Significance annotations are based on multiplicity-adjusted *p* values. ns, not significant; * *p*_adj < 0.05.

The candidate genes were evaluated by RT-qPCR in the independent four-group clinical cohort, comprising 30 participants per group. PBMCs were isolated, and the mRNA expression of CD4, MRPS21, and CRTC2 was detected using RT-qPCR; relative expression levels were calculated using the 2^−ΔΔCt^ method. CD4 expression differed significantly among the four groups and was higher in the FH-MDD group than in the MDD group (95% CI = 1.275–3.415, *p* = 0.004, Table 2, Figure 10). Importantly, this difference remained statistically significant after adjustment for BMI and other clinical covariates, including age, sex, WBC, C-reactive protein, HAMD-24, urea, creatinine, and uric acid (adjusted fold ratio = 1.788, 95% CI: 1.069–2.991, *p* = 0.027; Supplementary Table S4). In contrast, MRPS21 and CRTC2 did not show statistically significant adjusted differences between the FH-MDD and MDD groups (MRPS21: adjusted *p* = 0.117; CRTC2: adjusted *p* = 0.083). Given LDL-C is an intrinsic clinical feature of FH and was markedly separated between the MDD and FH-MDD groups, it was not included in the primary model. In a conservative sensitivity model that additionally included LDL-C, the effect estimate was attenuated and no longer statistically significant (adjusted fold ratio = 3.878, 95% CI: 0.790–19.032, *p* = 0.094; Supplementary Table S4). Therefore, the present analysis does not establish that the CD4 association is independent of LDL-C.

### 2.9. Flow-Cytometric Characterization of CD4+ T-Cell Subsets and Activation-Associated Phenotypes

**Figure 11:**
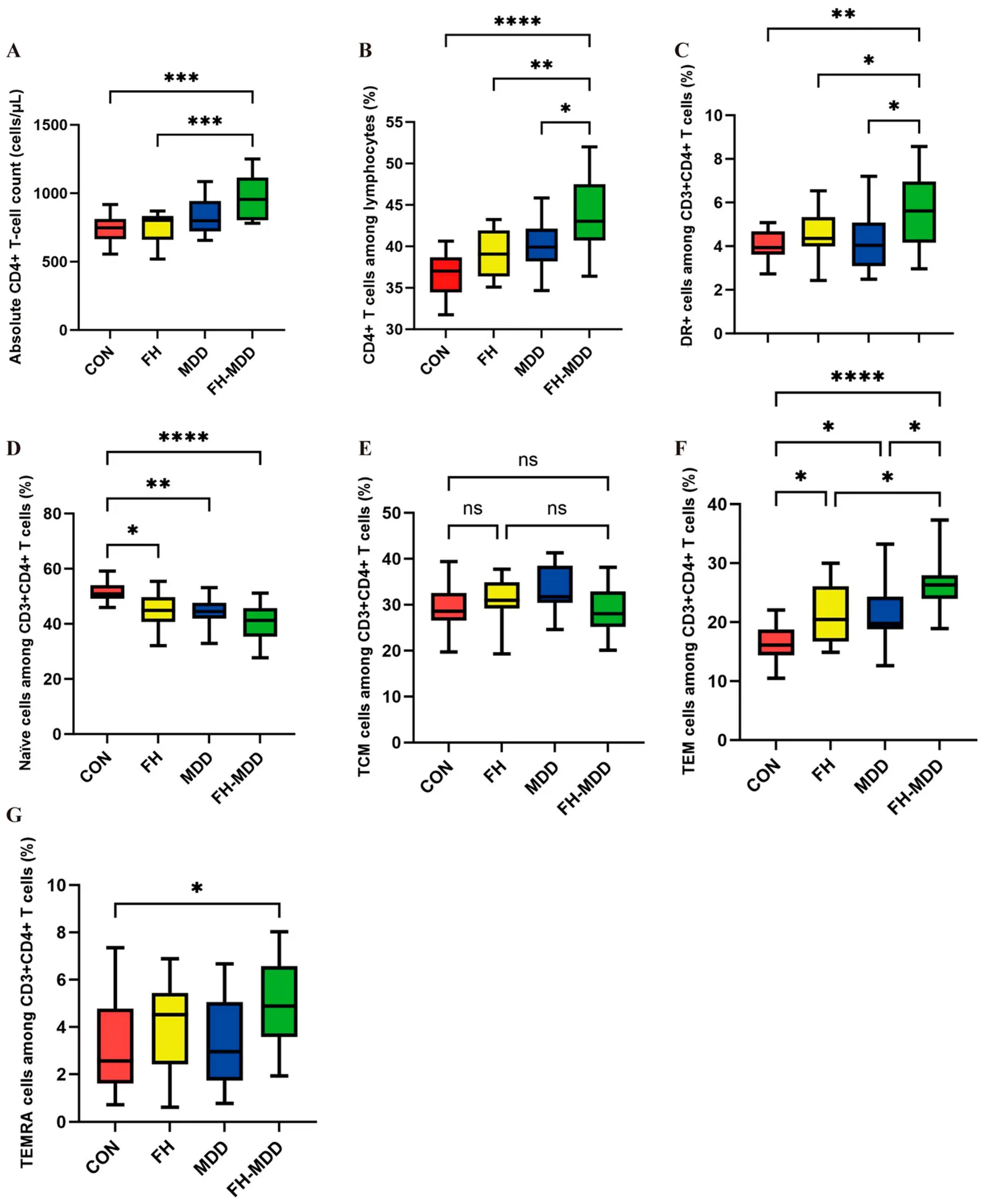
Flow-cytometric characterization of circulating CD4+ T-cell abundance, subset distribution, and activation-associated phenotypes in the four clinical groups. (**A**) Absolute CD3+CD4+ T-cell count. (**B**) Proportion of CD3+CD4+ T cells among lymphocytes. (**C**) Proportion of HLA-DR+ cells among CD3+CD4+ T cells. (**D**) Proportion of naïve CD4+ T cells, defined as CD45RA+CCR7+. (**E**) Proportion of central memory CD4+ T cells (TCM), defined as CD45RA−CCR7+. (**F**) Proportion of effector memory CD4+ T cells (TEM), defined as CD45RA−CCR7−. (**G**) Proportion of terminally differentiated effector memory CD4+ T cells re-expressing CD45RA (TEMRA), defined as CD45RA+CCR7−. Significance annotations are based on multiplicity-adjusted *p* values. ns, not significant, * *p*_adj < 0.05, ** *p*_adj < 0.01, *** *p*_adj < 0.001, and **** *p*_adj < 0.0001.

Flow-cytometric analysis included 48 participants, with 12 participants in each group. As shown in Table 3, the four groups were comparable in age, sex distribution, BMI, white blood cell count, C-reactive protein, urea, creatinine, and uric acid levels (all *p* > 0.05), whereas HAMD-24 scores and LDL-C levels differed as expected, according to MDD and FH status, respectively (both *p* < 0.05, Table 3). The absolute CD4+ T-cell count was significantly higher in the FH-MDD group than in the CON and FH groups (both *p* < 0.001), but did not differ significantly between the FH-MDD and MDD groups (Figure 11A). The proportion of CD4+ T cells among lymphocytes was significantly higher in the FH-MDD group than in the CON (*p* < 0.0001), FH (*p* < 0.01), and MDD (*p* < 0.05) groups (Figure 11B). These findings indicate that the CD4-related transcriptional signal in FH-MDD was accompanied by an altered circulating CD4+ T-cell profile.

The proportion of HLA-DR+ cells among CD3+CD4+ T cells was significantly higher in the FH-MDD group than in the CON (*p* < 0.01), FH (*p* < 0.05), and MDD (*p* < 0.05) groups (Figure 11C). The proportion of naïve CD4+ T cells was significantly lower in the FH, MDD, and FH-MDD groups than in the CON group (*p* < 0.05, *p* < 0.01, and *p* < 0.0001, respectively) (Figure 11D), whereas no significant differences in TCM proportions were observed among the groups (Figure 11E). In contrast, TEM proportions were significantly higher in the FH and MDD groups than in the CON group (both *p* < 0.05). The FH-MDD group showed a further increase in TEM proportions compared with the CON group (*p* < 0.0001), the FH group (*p* < 0.05), and the MDD group (*p* < 0.05) (Figure 11F). The TEMRA proportion was also significantly higher in the FH-MDD group than in the CON group (*p* < 0.05) (Figure 11G). Collectively, these findings indicate that FH-MDD co-occurrence was associated with an increased circulating CD3+CD4+ T-cell proportion, a shift from a naïve toward an effector-memory phenotype, and an increased HLA-DR+ activation-associated CD4+ T-cell profile.

## 3. Discussion

Familial hypercholesterolemia (FH) is a common inherited disorder characterized by markedly elevated plasma low-density lipoprotein cholesterol (LDL-C) levels. Beyond its cardiometabolic consequences, FH-related lipid dysregulation may also be associated with depressive symptoms, while depression frequently co-occurs with lipid abnormalities. However, the biological basis of FH-MDD co-occurrence remains poorly understood, and reliable molecular markers have not been established. In this study, we integrated bioinformatics, machine learning, immune-cell infiltration analysis, genome-wide association data, and summary-data-based Mendelian randomization to identify shared molecular signatures and construct an exploratory nomogram. Subsequent SMR analysis indicated that genetically predicted higher CD4 expression was inversely associated with MDD risk. Sensitivity meta-analysis further supported the cross-dataset consistency of CD4 and CRTC2, whereas MRPS21 showed discordant effects and substantial heterogeneity, further supporting the prioritization of CD4 as the most robust candidate. In contrast to the inverse association observed in SMR, RT-qPCR and flow-cytometric analyses showed increased CD4-related signals in patients with established FH-MDD, including higher CD4 mRNA expression and alterations in the circulating CD4+ T-cell compartment. These findings identify CD4 as an immune-related candidate across distinct analytical levels, but should not be interpreted as directionally concordant evidence.

Protein association analysis and machine-learning-based feature selection identified three candidate genes: MRPS21, CRTC2, and CD4. The exploratory diagnostic model showed discrimination in the training dataset and moderate performance in external validation. Single-gene GSEA indicated enrichment in T-cell receptor (TCR) signaling, nuclear mRNA degradation, and tumor necrosis factor-mediated signaling. TCR signaling is central to T-cell activation and immune responses. Activated T cells produce cytokines such as tumor necrosis factor-α (TNF-α), a major pro-inflammatory mediator, and TCR activation can promote TNF-α expression and secretion [14]. Because TNF-α mRNA is typically unstable and has a short half-life, rapid mRNA degradation helps prevent excessive or sustained inflammatory responses. CD4 encodes a T-cell co-receptor that participates in TCR signaling and influences T-cell activation and differentiation. CRTC2, as a transcriptional regulator, may affect the expression of genes involved in TCR and TNF-related signaling. These findings implicate TCR-related immune regulation in the biological overlap between FH and MDD. However, FH-related cholesterol dysregulation may also affect depression through non-inflammatory mechanisms involving neuronal membrane organization, myelin integrity, and serotonergic neurotransmission. In particular, cholesterol-rich membrane microdomains influence the localization and function of serotonin receptors and transporters and may therefore affect neural signaling and emotional regulation.

Immune dysregulation is increasingly recognized as an important contributor to the onset and progression of depression. Hypercholesterolemia is commonly accompanied by chronic low-grade inflammation, which is closely linked to depressive pathology [15]. In hypercholesterolemic mice, monocytes accumulate cholesterol esters, potentially altering their function and promoting a pro-inflammatory phenotype [16]. Monocytes from individuals with depression have also been reported to exhibit inflammatory activation and increased cytokine production [17]. Moreover, activated monocytes can release matrix metalloproteinase-8 (MMP8) and remodel the extracellular space of the nucleus accumbens, a process linked to stress-induced social avoidance behavior [18]. In the present study, CIBERSORT-based deconvolution suggested increased monocyte-related signals and reduced CD8+ T-cell and resting memory CD4+ T-cell signals in MDD samples, indicating a coordinated peripheral immune alteration. The flow-cytometric findings do not necessarily conflict with the CIBERSORT results. CIBERSORT estimated the relative transcriptomic signal of resting memory CD4+ T cells in MDD versus healthy controls, whereas flow cytometry directly quantified the overall proportion and absolute count of circulating CD4+ T cells across the four clinical groups. The two analyses therefore assessed different comparisons and different levels of CD4+ T-cell organization. In FH, chronic LDL-C overload may promote lipid accumulation, monocyte activation, cytokine release, and antigen-presenting activity [19]. Persistent lipid overload and low-grade inflammation may further drive immune-cell metabolic reprogramming, trained immunity, inflammasome activation, and innate–adaptive immune imbalance, which may partly explain the enrichment of TCR/CD4-related pathways observed in this study. These peripheral immune disturbances may also affect depression-related central mechanisms through endothelial activation, oxidative stress, vascular inflammation, blood–brain barrier disruption, and microglial activation, ultimately impairing hippocampal neurogenesis, synaptic plasticity, and monoaminergic regulation in susceptible individuals. Consistently, lower CD4+/CD8+ T-cell ratios, reduced albumin levels, and higher monocyte percentages have been associated with greater depression severity [20]. Therefore, immunoregulatory strategies targeting monocyte activation and T-cell imbalance may provide a potential direction for future mechanistic studies on the co-occurrence of FH and MDD.

CD4 is a surface co-receptor expressed mainly on helper T cells. It cooperates with the T-cell receptor (TCR) to recognize antigenic peptides presented by major histocompatibility complex class II molecules, thereby contributing to adaptive immune responses [21]. The role of T cells in depression has received increasing attention, although the findings remain heterogeneous [22]. Some studies have reported reduced CD4+ T-cell levels in patients with MDD, whereas others have observed increased circulating CD4+ T cells expressing interleukin-17 and TNF [21]. Increased T-cell activation and impaired regulatory T-cell function have also been associated with MDD and symptom severity [23]. In this study, resting memory CD4+ T-cell signals were lower in MDD samples than in healthy controls, suggesting an altered CD4+ T-cell-related immune profile. CD4+ T-cell heterogeneity has also been linked to antidepressant response. Recent single-cell RNA-sequencing and ATAC-sequencing analyses showed that naïve CD4+ T-cell differentiation patterns were associated with treatment response in MDD [22]. These findings suggest that CD4+ T-cell subsets may have potential value as immune-related biomarkers. In FH, chronically elevated LDL-C may promote vascular inflammation and immune-cell recruitment, including CD4+ T-cell infiltration [24]. CD4+ T-cell-associated cytokines, including IL-1β, IL-6, and TNF-α, may aggravate vascular inflammation and alter neuroimmune communication, potentially contributing to neuroinflammation and depressive symptoms [25]. Larger, well-controlled cohorts, detailed immune-cell subset profiling, and functional experiments are needed to determine whether the CD4-related signal reflects CD4-mediated T-cell regulation, systemic inflammation, pleiotropic immune effects, or shared genetic architecture affecting T-cell biology and susceptibility to depression.

Importantly, the clinical RT-qPCR and flow-cytometric analyses provided complementary evidence for the CD4-related alteration. In the PBMC cohort, CD4 mRNA expression was significantly higher in patients with FH-MDD than in patients with MDD alone, whereas MRPS21 and CRTC2 showed no significant adjusted differences. CD4 mRNA expression was higher in the FH-MDD group than in the MDD group, while flow cytometry showed an increased circulating CD3+CD4+ T-cell proportion in FH-MDD. These findings suggest that the increased CD4 transcript level in FH-MDD may, at least in part, be accompanied by an alteration in the circulating CD4+ T-cell compartment [20]. Notably, this disease-state elevation differs in direction from the SMR finding, in which genetically predicted higher CD4 expression was associated with lower MDD risk. These results likely reflect distinct biological contexts, because SMR evaluates genetically proxied expression in relation to disease susceptibility, whereas RT-qPCR and flow cy-tometry characterize peripheral immune features in individuals with established disease. Therefore, the increased CD4-related signal observed in FH-MDD may reflect dis-ease-state or secondary immune changes, including possible reverse causation, rather than an upstream effect that increases MDD susceptibility. The expanded flow-cytometric analysis further refined the CD4-related cellular phenotype. Naïve CD4+ T-cell proportions were reduced in all three disease groups, whereas TEM cells were increased in FH and MDD and were further elevated in FH-MDD compared with either single-disease group. Together with the absence of significant differences in TCM cells, these findings suggest a redistribution of the circulating CD4+ T-cell compartment from a naïve toward a more differentiated effector-memory phenotype, rather than a uniform expansion of all memory subsets [22]. This interpretation is consistent with the reported reduction in naïve CD4+ T cells in MDD and with previous studies linking lower naïve and higher memory or TEM CD4+ T-cell proportions to LDL levels and atherosclerotic burden [26]. Moreover, recent deep immunophenotyping has associated CD4+ TEM and TEMRA phenotypes with symptom severity and treatment outcomes in MDD [27].The increased proportion of HLA-DR+ cells among CD3+CD4+ T cells further indicates that the altered CD4+ T-cell compartment in FH-MDD was accompanied by an activation-associated phenotype. Previous studies have reported increased expression of T-cell HLA-DR in subsets of patients with depression, while experimental evidence suggests that HLA-DR can identify recently divided effector CD4+ T cells [28]. Nevertheless, HLA-DR expression alone is not functionally specific and is not confined exclusively to activated T cells. Accordingly, the present results provide subset-specific immunophenotypic evidence extending the CD4 transcriptional signal identified by RT-qPCR, but they do not establish enhanced cytokine production, proliferative capacity, antigen responsiveness, or a causal role for CD4+ T cells in FH-MDD pathogenesis.

In contrast, the non-significant changes in MRPS21 and CRTC2 do not necessarily exclude their potential involvement, but may indicate more subtle or context-dependent effects, or may reflect the limited sample size of the validation cohort [29]. MRPS21 encodes a mitochondrial ribosomal protein involved in mitochondrial translation and cellular energy metabolism. Altered MRPS21 expression could affect oxidative phosphorylation, ATP production, reactive oxygen species generation, and oxidative stress, potentially linking mitochondrial dysfunction, lipid dysregulation, immune activation, and depression. CRTC2 is a CREB-regulated transcriptional coactivator involved in energy homeostasis, gluconeogenesis, metabolic adaptation, and cellular stress responses. Dysregulated CRTC2 signaling may therefore affect metabolic flexibility and stress-related pathways, providing a possible connection between FH-related metabolic disturbances and MDD susceptibility [30]. Nevertheless, because MRPS21 and CRTC2 were not significantly validated by PBMC RT-qPCR, they should remain exploratory candidates rather than confirmed biomarkers. Overall, the convergent transcriptomic, RT-qPCR, and flow-cytometric findings support a preliminary association between FH-MDD co-occurrence and an altered peripheral CD4-related immune profile. The enrichment of TCR- and TNF-related pathways provides a possible biological context for this association but remains based primarily on computational inference. The proposed immunometabolic relationship should therefore be regarded as a working hypothesis rather than an established mechanistic model. Clinically, depressive symptoms may warrant greater attention in patients with FH alongside routine LDL-C management. Conversely, FH should be considered in patients with MDD, particularly those with obesity, elevated LDL-C, or a family history of hypercholesterolemia. Larger, well-controlled studies are needed to evaluate the clinical relevance of integrated lipid management, psychiatric care, and immune-related monitoring.

Bioinformatic findings on FH-MDD co-occurrence also support greater integration at the clinical-practice and public-health levels. Lipid-lowering treatments for FH, including statins and PCSK9 inhibitors, and antidepressants such as SSRIs and SNRIs are generally prescribed separately, while guidance on coordinated management remains limited [31,32,33]. Future studies may explore therapies with both lipid-modulating and neuroprotective effects and develop individualized management strategies based on clinical and molecular profiles. Patients with FH often require long-term or lifelong medication, and depression-related reductions in motivation and adherence may compromise lipid control, potentially creating a “metabolism–mood” cycle [34]. Therefore, incorporating depression screening and psychological support into FH care pathways, as well as routinely assessing lipid profiles in psychiatric settings, may help identify patients with overlapping metabolic and depressive risks and support more integrated intervention strategies.

This study still has certain limitations: First, the transcriptomic data were mainly derived from peripheral blood, which may not fully reflect molecular alterations in the central nervous system. Second, because few genes remained significant after dataset-specific FDR correction, a nominal *p*-value threshold was used for the initial exploratory screening. A subsequent sensitivity meta-analysis across the two FH datasets strengthened the cross-dataset support for CD4 and CRTC2, whereas MRPS21 showed substantial heterogeneity. Although these results improved the robustness of candidate prioritization, the downstream PPI, enrichment, machine-learning, and nomogram analyses remain exploratory and require further independent validation. Third, the RT-qPCR validation cohort was small, limiting the power of biomarker validation and sex-stratified analyses. CD4 showed the most extensive support across analyses, but the clinical association was attenuated after LDL-C adjustment; therefore, CD4 should be regarded as a candidate immune-related signature rather than an independent biomarker. Although the nomogram performed well in the training dataset, external validation showed only moderate discrimination. The lower external AUC may reflect differences in sample sources, platforms, batch effects, cohort composition, and phenotype definitions. The nomogram should therefore be considered exploratory and hypothesis-generating until validated in larger independent cohorts. Finally, lipidomic, metabolomic, and microbiome data were unavailable. The roles of lipid metabolic and gut microbiota-related pathways, as well as the specificity of the candidate markers, should therefore be interpreted cautiously. Lipidomic and metabolomic analyses may characterize downstream metabolic changes, but microbiome profiling is required to directly assess gut microbiota-related pathways. Because comparator datasets from other metabolic or inflammatory disorders were not included, CD4, MRPS21, and CRTC2 should be regarded as candidate shared markers associated with FH-MDD co-occurrence rather than markers unique to FH-associated depression.

## 4. Materials and Methods

The analytical approach is outlined in Figure 1. Three discovery datasets were used, including GSE6054 and GSE13985 for FH and GSE98793 for MDD. GSE19738 was used as an external validation dataset (https://www.ncbi.nlm.nih.gov/geo). The FH expression datasets (GSE6054, GSE13985) comprised peripheral blood samples from 15 FH patients and 18 healthy controls. The MDD expression dataset included peripheral blood samples from 64 healthy controls and 128 MDD patients. R software (R 4.4.2) was used to integrate sample gene data across each dataset. The LIMMA package screened for DEGs between disease and healthy control groups, setting thresholds of absolute log2 fold change (log2FC) > 0.585 and *p*-value < 0.05. To improve statistical transparency, *p* values were additionally adjusted using the Benjamini–Hochberg false discovery rate (FDR) method. Because very few or no genes remained significant after FDR correction in some datasets, genes selected using the *p* value threshold were used only for exploratory integrative screening and candidate prioritization rather than confirmatory inference. The intersection of DEGs across datasets was identified as co-disease genes. The expression patterns of DEGs were visualized using the pheatmap and ggplot2 packages to generate heatmaps and volcano plots, respectively. To assess cross-dataset consistency of the initially identified FH-associated genes, an inverse-variance-weighted fixed-effect meta-analysis was performed on intersecting genes. Benjamini–Hochberg correction was applied to meta-analysis *p* values within the candidate set, and between-study heterogeneity was evaluated using Cochran’s Q test and the I^2^ statistic.

### 4.1. Identification of Key Modules and Functional Genes

To characterize the biological relationships among FH- and MDD-related genes, Gene Ontology (GO) enrichment analysis was performed using the R package clusterProfiler (R software (R 4.4.2)). GO terms were analyzed across three categories: biological process (BP), cellular component (CC), and molecular function (MF). GO and KEGG enrichment analyses were performed to investigate the biological functions and pathways of the candidate genes. *p* values were adjusted for multiple testing using the Benjamini–Hochberg false discovery rate (FDR) method, and terms or pathways with FDR-adjusted *p* < 0.05 were considered statistically significant. Spearman correlation analysis was performed to evaluate associations among key FH- and MDD-related genes. The cowplot package (R software (R 4.4.2)) was used to visualize the results through heatmaps, scatter plots, and correlation curves. A *p* value < 0.05 was regarded as statistically significant. Protein–protein interaction (PPI) analysis was conducted using the STRING database with a medium confidence score threshold > 0.4 [35]. The resulting PPI network was visualized in Cytoscape (v3.7.1). The MCODE plugin was then applied to identify densely connected network modules using the following parameters: Degree Cutoff = 2, Haircut = true, Node Score Cutoff = 0.2, k-core = 2, and Max Depth = 100. The two highest-scoring modules were retained for subsequent analyses.

### 4.2. Weighted Gene Co-Expression Network Analysis (WGCNA)

WGCNA was used to identify gene modules showing coordinated expression patterns and to evaluate their relationships with disease phenotypes [36]. Based on the peripheral blood expression dataset GSE98793, the WGCNA package (R software (R 4.4.2)) was used to construct a scale-free co-expression network for MDD. The top 25% of genes ranked by expression level were selected for WGCNA. A scale-free topology fitting index of R^2^ = 0.9 and a soft-thresholding power of β = 10 were used to ensure that the network approximated scale-free topology. Gene modules were identified through dynamic tree cutting and topological overlap matrix (TOM)-based hierarchical clustering, with the minimum module size set to 100. Modules most strongly associated with MDD were selected for further analysis.

### 4.3. Machine Learning-Based Feature Selection and Exploratory Nomogram Construction

To identify candidate feature genes for the co-occurrence of FH and MDD, the 54 shared candidate genes obtained from the integrative analysis were entered into two complementary machine-learning algorithms. The GSE98793 expression matrix was used as the training dataset for feature selection and model construction, whereas GSE19738 was used only as an independent external validation dataset and was not involved in feature selection or model fitting. In both datasets, MDD patients were defined as the positive class and healthy controls as the negative class. LASSO logistic regression was performed using the glmnet package to reduce model complexity and select informative genes [37]. The penalty parameter λ was selected according to the model-selection procedure implemented in glmnet, and λ = 0.03 was used for feature selection. Random Forest analysis was performed using the randomForest package with 500 trees, and genes with MeanDecreaseGini > 2 were retained as candidate features [38]. The overlapping genes identified by both LASSO and Random Forest were considered the final candidate feature genes.

The selected genes were then included in a multivariable logistic regression model to construct an exploratory nomogram using the rms package (R software (R 4.4.2)) [39]. Model discrimination was evaluated by ROC curves and AUC values using the pROC package. Calibration was assessed using calibration curves and the Hosmer–Lemeshow goodness-of-fit test, and potential clinical net benefit was explored using decision curve analysis. External validation was performed in the independent GSE19738 dataset by applying the same selected feature genes to evaluate model discrimination. Given the limited sample size and heterogeneity among public datasets, the nomogram was considered exploratory and hypothesis-generating rather than a model ready for direct clinical application.

### 4.4. Immune Infiltration Analysis

The relative abundances of 22 immune cell types in disease samples were estimated using the CIBERSORT algorithm [40]. The Wilcoxon rank-sum test was used to compare differences in immune cell proportions between MDD samples and healthy control samples; *p* < 0.05 was considered statistically significant. The distribution patterns of immune cells were visualized using the ggplot2 package (R software (R 4.4.2)). A Spearman correlation analysis was further conducted to investigate the relationship between the expression of diagnostic biomarkers and immune cell composition. Correlations were considered statistically significant if *p* < 0.05.

### 4.5. GSEA

In the MDD dataset GSE98793, gene set enrichment analysis was performed for each feature gene using the org.Hs.eg.db (R software (R 4.4.2)) and cluster Profiler packages to identify differentially enriched signaling pathways between the MD group and healthy controls. The EnrichPlot package (R software (R 4.4.2)) was used to visualize the top five activated and suppressed pathways in MDD.

### 4.6. SMR Analysis

We retrieved 170,756 MDD cases and 329,443 controls, with 8,483,301 genetic variants from the Psychiatric Genome Consortium (https://pgc.unc.edu/, accessed on 10 November 2025) ebi-a-GCST90086060 database, which collected GWAS summary statistics from 170,756 MDD cases and 329,443 controls (totaling 8,483,301 genetic variants). This consortium represents one of the largest, most innovative, and productive platforms in the field of psychiatry [41]. Diagnostic criteria for MDD are detailed in the source data. Single-nucleotide polymorphisms (SNPs) associated with expression quantitative trait loci (eQTLs) were selected as instrumental variables (IVs) to evaluate genetically supported associations between gene expression and MDD risk. Summary statistics for whole-blood eQTLs across 15,882 genes were obtained from 31,684 individuals in the eQTLGen database [42]. The current study focuses exclusively on cis-eQTLs located within 1 Mb of the gene start or end points.

### 4.7. Sensitivity Analysis

We assessed the strength of SNPs used as instruments via F-statistics, including only those with F-statistics > 10 to minimize weak instrument bias [43]. The instrument-dependent (HEIDI) test in SMR v1.3.1 was applied to distinguish pleiotropy from linkage. Among all instrumental variables, HEIDI *p*-values < 0.1 indicated significant heterogeneity.

### 4.8. Sample Collection of the Four-Group Clinical Cohort

This study included patients with major depressive disorder (MDD) who were treated at the Department of Psychiatry of the First Affiliated Hospital of Chongqing Medical University between August 2025 and March 2026. The clinical validation cohort consisted of four groups: healthy controls (CON, n = 30), FH patients without MDD (FH, n = 30), MDD patients without FH (MDD, n = 30), and patients with co-occurring FH and MDD (FH-MDD, n = 30), for a total of 120 participants. MDD was diagnosed according to DSM-5 criteria, and patients with HAMD-24 scores > 24 were considered to have moderate-to-severe depressive symptoms. FH was diagnosed based on a family history of familial hypercholesterolemia combined with significantly elevated serum LDL-C levels, defined as LDL-C levels > 4.9 mmol/L in adults and >3.5 mmol/L in children. The shorthand label “FH-MDD” is merely a grouping marker for statistical analysis, which exclusively describes patients receiving concurrent clinical diagnoses of familial hypercholesterolemia (FH) and major depressive disorder (MDD). Patients with a HAMD-24 score > 24 were diagnosed with MDD. Participants with other psychiatric disorders, acute infection, severe inflammatory disease, autoimmune disease, severe hepatic or renal dysfunction, or recent antidepressant use within two weeks were excluded. This clinical study has been approved and registered by the Ethics Committee of the First Affiliated Hospital of Chongqing Medical University, under number 2020-856.

### 4.9. Isolation and Preservation of Peripheral Blood Mononuclear Cells (PBMCs)

Peripheral venous blood was collected into anticoagulated tubes and processed at room temperature as soon as possible after collection. Whole blood was gently mixed and diluted 1:1 with calcium- and magnesium-free PBS. The diluted blood was then carefully layered onto Ficoll-Paque density gradient medium, avoiding disruption of the gradient. Samples were centrifuged at 400× *g* for 30 min at room temperature without brake. After centrifugation, distinct layers were observed, including the plasma layer, PBMC layer, Ficoll layer, and red blood cell layer. The PBMC layer at the plasma–Ficoll interface was carefully aspirated using a sterile pipette and transferred to a new centrifuge tube. PBS was then added to wash the cells, followed by centrifugation at 300× *g* for 10 min. The washing step was repeated twice to remove residual platelets, plasma proteins, and Ficoll. After the final wash, the supernatant was discarded, and TRIzol reagent was added to the PBMC pellet for complete lysis. The lysate was transferred to an RNase-free cryovial and stored at −80 °C.

### 4.10. RT-qPCR

Total RNA was extracted from PBMC samples using the TRIzol method according to the manufacturer’s instructions. RNA concentration and purity were assessed by UV spectrophotometry, and RNA quality was evaluated using the A260/A280 ratio. Samples with insufficient RNA concentration, poor RNA purity, or incomplete clinical information were excluded from subsequent RT-qPCR analysis. Equal amounts of total RNA were reverse-transcribed into cDNA using PrimeScript™ RT Master Mix (RR036A, Takara, Japan). Quantitative PCR was performed using TB Green^®^ Premix Ex Taq™ II (Tli RNaseH Plus; RR820A, Takara, Japan) on a real-time PCR system (LightCycler 480, Roche Diagnostic, Indianapolis, IN, USA). The qPCR cycling conditions were as follows: initial denaturation at 95 °C for 30 s, followed by 40 cycles of 95 °C for 5 s and 60 °C for 30 s. A melting curve analysis was performed to confirm amplification specificity. GAPDH was used as the internal reference gene. Each qPCR reaction was performed with three technical replicates, and three independent biological replicates were included to ensure reproducibility. Relative mRNA expression levels of CD4, MRPS21, and CRTC2 were calculated using the 2^−ΔΔCt^ method. Primer sequences are listed in Supplementary Table S5.

### 4.11. Flow-Cytometric Analysis of CD4+ T Cells

Flow-cytometric analysis was performed in 48 participants, with 12 participants in each of the CON, FH, MDD, and FH-MDD groups. PBMCs were stained with fluorophore-conjugated antibodies against CD3, CD4, CD45RA, CCR7, and HLA-DR. After exclusion of debris and doublets, lymphocytes were identified according to forward- and side-scatter characteristics. CD3+ T cells were identified, and CD4+ T cells were subsequently defined within the CD3+ population.CD3+CD4+ T cells were further classified as naïve cells (CD45RA+CCR7+), TCM cells (CD45RA−CCR7+), TEM cells (CD45RA−CCR7−), and TEMRA cells (CD45RA+CCR7−). HLA-DR expression was quantified as the proportion of HLA-DR+ cells among total CD3+CD4+ T cells. Data were acquired using a Sony SA3800 Spectral Cell Analyzer and analyzed using a standardized gating strategy. Detailed antibody information and instrument settings are provided in Supplementary Tables S6 and S7.

### 4.12. Statistical Analysis

Continuous variables across four clinical groups were compared by one-way ANOVA or the Kruskal–Wallis test according to data distribution. Homogeneity of variances was assessed using the Brown–Forsythe test. For variables satisfying the ho-mogeneity-of-variance assumption, ordinary one-way ANOVA was followed by Tukey’s multiple-comparisons test for post hoc pairwise comparisons. For variables showing significant heterogeneity of variances, Welch’s ANOVA followed by Dunnett’s T3 mul-tiple-comparisons test was used. When nonparametric analysis was required, the Krus-kal–Wallis test was followed by Dunn’s multiple-comparisons test. The multiplici-ty-adjusted P values generated by the corresponding post hoc procedures were used for pairwise statistical inference and for the significance annotations in Figure 10 and Figure 11. Categorical variables were analyzed using the chi-square test or Fisher’s exact test.

For RT-qPCR validation, relative gene expression levels calculated via the 2^−ΔΔCt^ method were log2-transformed prior to regression-based statistical analysis. Differences in candidate gene expression among groups were evaluated by multiple linear regression/analysis of covariance (ANCOVA) after adjusting for potential confounders. The base adjustment model included age, sex, body mass index (BMI), white blood cell count (WBC), C-reactive protein, 24-item Hamilton Depression Rating Scale (HAMD-24) score, urea, creatinine and uric acid as covariates. An additional sensitivity analysis model was constructed with further adjustment for low-density lipoprotein cholesterol (LDL-C). Adjusted results are detailed in Supplementary Table S4. In all tests, a *p*-value < 0.05 was considered statistically significant. Causal inference between genome-wide association studies (GWAS) and cis-eQTLs was evaluated using the SMR multi-functional tool. The most highly correlated cis-QTLs were identified by screening gene windows centered on the corresponding gene (±1000 kb) with a *p*-value threshold < 5.0 × 10^−8^. Across all datasets (including linkage disequilibrium [LD] reference samples, QTL aggregate data, and phenotype aggregate data), SNPs with allele frequency differences exceeding a specified threshold (0.2 in this study) were excluded.

## 5. Conclusions

This study identified candidate genes associated with the co-occurrence of FH and MDD by integrating transcriptomic analysis, machine learning, SMR analysis, and clinical validation. CD4 showed the most consistent support across analyses, with the RT-qPCR and flow-cytometric results providing additional experimental support, whereas MRPS21 and CRTC2 should be interpreted as exploratory candidates requiring further validation. The exploratory nomogram showed good discrimination in the training dataset but only moderate performance in external validation. Overall, these findings provide preliminary evidence for shared CD4-related immune alterations between FH and MDD; however, the current evidence remains associative, and further functional validation is required.

## Figures and Tables

**Table 1:** Comparison of basic demographic and clinical characteristics among the four clinical groups.

	CON	FH	MDD	FH-MDD	*p*-Value
N	30	30	30	30	
Age (years)	20.3 ± 3.3	20.9 ± 3.7	20.2 ± 3.7	19.3 ± 4.0	0.419
Gender (Female, n (%))	20 (66.67%)	20 (66.67%)	20 (66.67%)	20 (66.67%)	1.000
BMI (kg/m^2^)	24.8 ± 3.9	25.2 ± 3.6	23.5 ± 3.7	25.8 ± 4.3	0.137
WBC (×10^9^/L)	7.13 ± 1.47	7.63 ± 1.39	7.27 ± 1.58	8.02 ± 1.96	0.143
C-reactive protein (mg/L)	4.3 ± 1.2	4.6 ± 1.1	4.8 ± 1.0	4.7 ± 0.8	0.216
HAMD-24	3.6 ± 1.9	3.8 ± 1.8	30.7 ± 2.6	30.3 ± 2.4	<0.001
Urea (mmol/L)	4.4 ± 0.8	4.9 ± 0.8	4.6 ± 1.2	4.4 ± 0.9	0.104
Creatinine (μmol/L)	68.6 ± 14.0	68.9 ± 11.5	72.4 ± 14.3	69.4 ± 13.0	0.671
Uric acid (μmol/L)	333.0 ± 61.6	300.6 ± 61.7	336.3 ± 95.3	312.6 ± 52.6	0.154
LDL-C (mmol/L)	2.66 ± 0.34	5.45 ± 0.45	2.59 ± 0.24	5.61 ± 0.33	<0.001

Data are presented as mean ± SD, median (IQR), or n (%), as appropriate. Overall between-group comparisons were performed using one-way ANOVA, Kruskal–Wallis test, chi-square test, or Fisher’s exact test, as appropriate. BMI, body mass index; WBC, white blood cell count; HAMD-24, 24-item Hamilton Depression Rating Scale; LDL-C, low-density lipoprotein cholesterol.

**Table 2:** RT-qPCR validation of candidate biomarkers in the four-group clinical cohort.

Gene	CON Mean (SD)	FH Mean (SD)	MDD Mean (SD)	FH-MDD Mean (SD)	95% CI	*p* Value
CD4	0.720 (0.267)	1.380 (0.530)	1.001 (1.076)	1.789 (1.609)	2.086 (1.275–3.415)	0.004
MRPS21	1.703 (0.924)	1.606 (1.032)	2.255 (2.238)	1.505 (1.096)	0.733 (0.508–1.057)	0.180
CRTC2	2.150 (1.986)	2.645 (1.954)	3.002 (4.676)	3.460 (4.488)	1.720 (0.856–3.457)	0.530

Data are presented as mean (SD). Adjusted comparisons between FH-MDD and MDD were estimated using multivariable linear regression/ANCOVA based on log2-transformed 2^−ΔΔCt^ values, adjusted for age, sex, BMI, WBC, C-reactive protein, HAMD-24, urea, creatinine, and uric acid.

**Table 3:** Comparison of demographic and clinical characteristics among the four groups included in the flow-cytometric analysis.

	CON	FH	MDD	FH-MDD	*p*-Value
N	12	12	12	12	
Age (years)	20.6 ± 2.7	20.7 ± 4.8	21.1 ± 2.9	19.9 ± 3.2	0.875
Gender (Female, n (%))	7 (58.33%)	9 (75.00%)	8 (66.67%)	8 (66.67%)	0.861
BMI (kg/m^2^)	24.5 ± 3.4	24.7 ± 3.7	25.1 ± 3.2	22.0 ± 1.3	0.066
WBC (×10^9^/L)	7.54 ± 1.32	7.67 ± 1.35	7.12 ± 1.29	8.30 ± 1.31	0.188
C-reactive protein (mg/L)	4.5 ± 1.3	4.6 ± 0.9	4.5 ± 0.8	4.7 ± 0.6	0.950
HAMD-24	3.1 ± 1.4	4.4 ± 2.2	30.6 ± 2.1	30.0 ± 1.1	<0.001
Urea (mmol/L)	4.5 ± 1.0	4.6 ± 1.0	4.5 ± 0.8	4.5 ± 0.3	0.970
Creatinine (μmol/L)	69.5 ± 15.5	68.4 ± 12.9	74.6 ± 5.5	68.2 ± 7.6	0.463
Uric acid (μmol/L)	318.2 ± 52.8	318.3 ± 59.5	323.4 ± 76.6	301.2 ± 41.5	0.808
LDL-C (mmol/L)	2.65 ± 0.39	5.45 ± 0.41	2.54 ± 0.15	5.57 ± 0.16	<0.001

Data are presented as mean ± SD, median (IQR), or n (%), as appropriate. Overall between-group comparisons were performed using one-way ANOVA, Kruskal-Wallis test, chi-square test, or Fisher’s exact test, as appropriate. BMI, body mass index; WBC, white blood cell count; HAMD-24, 24-item Hamilton Depression Rating Scale; LDL-C, low-density lipoprotein cholesterol.

## Data Availability

The public datasets analyzed in this study are available in the GEO database (https://www.ncbi.nlm.nih.gov/geo/, accessed on 10 November 2025) under accession numbers GSE6054, GSE13985, GSE98793, and GSE19738. GWAS summary statistics for major depressive disorder were obtained from the Psychiatric Genomics Consortium (https://pgc.unc.edu/)/IEU OpenGWAS database under accession number ebi-a-GCST90086060. Whole-blood cis-eQTL summary statistics were obtained from the eQTLGen database (https://www.eqtlgen.org/). The data presented in this study are available on request from the corresponding author (the data are not publicly available due to privacy and ethical restrictions).

## Supplementary Materials

The following supporting information can be downloaded at https://www.mdpi.com/article/10.3390/ijms27177532/s1.

## References

[1] Marx W., Penninx B.W.J.H., Solmi M., Furukawa T.A., Firth J., Carvalho A.F., Berk M. (2023). Major depressive disorder. Nat. Rev. Dis. Primers.

[2] Herrman H., Patel V., Kieling C., Berk M., Buchweitz C., Cuijpers P., Furukawa T.A., Kessler R.C., Kohrt B.A., Maj M. (2022). Time for united action on depression: A Lancet-World Psychiatric Association Commission. Lancet.

[3] Luo K., Zhang M., Tu Q., Li J., Wang Y., Wan S., Li D., Qian Q., Xia L. (2025). From gut inflammation to psychiatric comorbidity: Mechanisms and therapies for anxiety and depression in inflammatory bowel disease. J. Neuroinflamm..

[4] Cipriani A., Furukawa T.A., Salanti G., Chaimani A., Atkinson L.Z., Ogawa Y., Leucht S., Ruhe H.G., Turner E.H., Higgins J.P.T. (2018). Comparative efficacy and acceptability of 21 antidepressant drugs for the acute treatment of adults with major depressive disorder: A systematic review and network meta-analysis. Lancet.

[5] Bianconi V., Banach M., Pirro M. (2021). Why patients with familial hypercholesterolemia are at high cardiovascular risk? Beyond LDL-C levels. Trends. Cardiovasc. Med..

[6] Dharmayat K.I., Vallejo-Vaz A.J., Stevens C.A.T., Brandts J.M., Lyons A.R.M., Groselj U., Abifadel M., Aguilar-Salinas C.A., Alhabib K., Alkhnifsawi M. (2023). Familial hypercholesterolaemia in children and adolescents from 48 countries: A cross-sectional study. Lancet.

[7] Chang N.-T., Su T.-C. (2019). Investigating the association between familial hypercholesterolemia and perceived depression. Atheroscler. Suppl..

[8] Chan M.F., Ganesh A., Mahadevan S., Shamli S.A., Al-Waili K., Al-Mukhaini S., Al-Rasadi K., Al-Adawi S. (2022). A Comprehensive Neuropsychological Study of Familial Hypercholesterolemia and Its Relationship with Psychosocial Functioning: A Biopsychosocial Approach. Brain Sci..

[9] Engel D.F., de Oliveira J., Lopes J.B., Santos D.B., Moreira E.L.G., Farina M., Rodrigues A.L.S., Brocardo P.d.S., de Bem A.F. (2016). Is there an association between hypercholesterolemia and depression? Behavioral evidence from the LDLr −/− mouse experimental model. Behav. Brain Res..

[10] Engel D.F., Grzyb A.N., Oliveira J., Pötzsch A., Walker T.L., Brocardo P.S., Kempermann G., Bem A.F. (2019). Impaired adult hippocampal neurogenesis in a mouse model of familial hypercholesterolemia: A role for the LDL receptor and cholesterol metabolism in adult neural precursor cells. Mol. Metab..

[11] Tyrovolas S., Lionis C., Zeimbekis A., Bountziouka V., Micheli M., Katsarou A., Papairakleous N., Metallinos G., Makri K., Polychronopoulos E. (2009). Increased body mass and depressive symptomatology are associated with hypercholesterolemia, among elderly individuals; results from the MEDIS study. Lipids Health Dis..

[12] Maes M., Almulla A.F., You Z., Zhang Y. (2025). Neuroimmune, metabolic and oxidative stress pathways in major depressive disorder. Nat. Rev. Neurol..

[13] Claudia M., Demis A.K., Jana V., John H., Nicholas W.W., Patrick A.L., Raffaele F. (2018). Genome, transcriptome and proteome: The rise of omics data and their integration in biomedical sciences. Brief. Bioinform..

[14] Weerakoon H., Mohamed A., Wong Y., Chen J., Senadheera B., Haigh O., Watkins T.S., Kazakoff S., Mukhopadhyay P., Mulvenna J. (2024). Integrative temporal multi-omics reveals uncoupling of transcriptome and proteome during human T cell activation. npj Syst. Biol. Appl..

[15] Bai Y., Cai Y., Chang D., Li D., Huo X., Zhu T. (2024). Immunotherapy for depression: Recent insights and future targets. Pharmacol. Ther..

[16] Fernandez-Ruiz I., Puchalska P., Narasimhulu C.A., Sengupta B., Parthasarathy S. (2016). Differential lipid metabolism in monocytes and macrophages: Influence of cholesterol loading. J. Lipid. Res..

[17] Nazimek K., Strobel S., Bryniarski P., Kozlowski M., Filipczak-Bryniarska I., Bryniarski K. (2017). The role of macrophages in anti-inflammatory activity of antidepressant drugs. Immunobiology.

[18] Cathomas F., Lin H.-Y., Chan K.L., Li L., Parise L.F., Alvarez J., Cuttoli R.D.-D., Aubry A.V., Muhareb S., Desland F. (2024). Circulating myeloid-derived MMP8 in stress susceptibility and depression. Nature.

[19] Chen J., Huang Y., Li X. (2024). The association between lymphocyte to high-density lipoprotein ratio and depression: Data from NHANES 2015-2018. Brain Behav..

[20] Zhou D., Yu H., Yao H., Yuan S., Xia Y., Huang L., Shen Y., Zhang J., Chen W. (2022). A novel joint index based on peripheral blood CD4+/CD8+ T cell ratio, albumin level, and monocyte count to determine the severity of major depressive disorder. BMC Psychiatry.

[21] Alvarez-Mon M.A., Gómez-Lahoz A.M., Orozco A., Lahera G., Diaz D., Ortega M.A., Albillos A., Quintero J., Aubá E., Monserrat J. (2021). Expansion of CD4 T Lymphocytes Expressing Interleukin 17 and Tumor Necrosis Factor in Patients with Major Depressive Disorder. J. Pers. Med..

[22] Sun Z., Zhang B., Zhou J., Luo Y., Zhu X., Wang Y., He Y., Zheng P., Zhang L., Yang J. (2024). Integrated Single-Cell RNA-seq and ATAC-seq Reveals Heterogeneous Differentiation of CD4+ Naive T Cell Subsets is Associated with Response to Antidepressant Treatment in Major Depressive Disorder. Adv. Sci..

[23] Rachayon M., Jirakran K., Sodsai P., Sughondhabirom A., Maes M. (2024). T cell activation and deficits in T regulatory cells are associated with major depressive disorder and severity of depression. Sci. Rep..

[24] Taghizadeh E., Taheri F., Hayat S.M.G., Montecucco F., Carbone F., Rostami D., Montazeri A., Sahebkar A. (2019). The atherogenic role of immune cells in familial hypercholesterolemia. IUBMB Life.

[25] Wolf D., Ley K. (2019). Immunity and Inflammation in Atherosclerosis. Circ. Res..

[26] Ammirati E., Cianflone D., Vecchio V., Banfi M., Vermi A.C., De Metrio M., Grigore L., Pellegatta F., Pirillo A., Garlaschelli K. (2012). Effector Memory T cells Are Associated With Atherosclerosis in Humans and Animal Models. J. Am. Heart Assoc..

[27] Stolfi F., Brasso C., Raineri D., Landra V., Mazzucca C.B., Ghazanfar A., Scotti L., Sinella R., Villari V., Cappellano G. (2025). Deep immunophenotyping of circulating immune cells in major depressive disorder patients reveals immune correlates of clinical course and treatment response. Brain Behav. Immun. Health.

[28] Tippalagama R., Singhania A., Dubelko P., Lindestam Arlehamn C.S., Crinklaw A., Pomaznoy M., Seumois G., deSilva A.D., Premawansa S., Vidanagama D. (2021). HLA-DR Marks Recently Divided Antigen-Specific Effector CD4 T Cells in Active Tuberculosis Patients. J. Immunol..

[29] Dentin R., Liu Y., Koo S.-H., Hedrick S., Vargas T., Heredia J., Yates J., Montminy M. (2007). Insulin modulates gluconeogenesis by inhibition of the coactivator TORC2. Nature.

[30] Cavdar Koc E., Burkhart W., Blackburn K., Moseley A., Spremulli L.L. (2001). The small subunit of the mammalian mitochondrial ribosome. Identification of the full complement of ribosomal proteins present. J. Biol. Chem..

[31] Berk M., Köhler-Forsberg O., Turner M., Penninx B.W.J.H., Wrobel A., Firth J., Loughman A., Reavley N.J., McGrath J.J., Momen N.C. (2023). Comorbidity between major depressive disorder and physical diseases: A comprehensive review of epidemiology, mechanisms and management. World Psychiatry.

[32] Köhler-Forsberg O., Otte C., Gold S.M., Østergaard S.D. (2020). Statins in the treatment of depression: Hype or hope?. Pharmacol. Ther..

[33] Wei Y.-G., Cai D.-B., Liu J., Liu R.-X., Wang S.-B., Tang Y.-Q., Zheng W., Wang F. (2020). Cholesterol and triglyceride levels in first-episode patients with major depressive disorder: A meta-analysis of case-control studies. J. Affect. Disord..

[34] Langslet G., Johansen A.K., Bogsrud M.P., Narverud I., Risstad H., Retterstøl K., Holven K.B. (2021). Thirty percent of children and young adults with familial hypercholesterolemia treated with statins have adherence issues. Am. J. Prev. Cardiol..

[35] Marteau P.-F. (2009). Time warp edit distance with stiffness adjustment for time series matching. IEEE Trans. Pattern. Anal. Mach. Intell..

[36] Hu C., Ge M., Liu Y., Tan W., Zhang Y., Zou M., Xiang L., Song X., Guo H. (2024). From inflammation to depression: Key biomarkers for IBD-related major depressive disorder. J. Transl. Med..

[37] Engebretsen S., Bohlin J. (2019). Statistical predictions with glmnet. Clin. Epigenet..

[38] Alderden J., Pepper G.A., Wilson A., Whitney J.D., Richardson S., Butcher R., Jo Y., Cummins M.R. (2018). Predicting Pressure Injury in Critical Care Patients: A Machine-Learning Model. Am. J. Crit. Care.

[39] Zhu E., Shu X., Xu Z., Peng Y., Xiang Y., Liu Y., Guan H., Zhong M., Li J., Zhang L.-Z. (2023). Screening of immune-related secretory proteins linking chronic kidney disease with calcific aortic valve disease based on comprehensive bioinformatics analysis and machine learning. J. Transl. Med..

[40] Xu J., Zhou H., Cheng Y., Xiang G. (2022). Identifying potential signatures for atherosclerosis in the context of predictive, preventive, and personalized medicine using integrative bioinformatics approaches and machine-learning strategies. EPMA J..

[41] Howard D.M., Adams M.J., Clarke T.-K., Hafferty J.D., Gibson J., Shirali M., Coleman J.R.I., Hagenaars S.P., Ward J., Wigmore E.M. (2019). Genome-wide meta-analysis of depression identifies 102 independent variants and highlights the importance of the prefrontal brain regions. Nat. Neurosci..

[42] Võsa U., Claringbould A., Westra H.-J., Bonder M.J., Deelen P., Zeng B., Kirsten H., Saha A., Kreuzhuber R., Yazar S. (2021). Large-scale cis- and trans-eQTL analyses identify thousands of genetic loci and polygenic scores that regulate blood gene expression. Nat. Genet..

[43] Burgess S., Thompson S.G., CRP CHD Genetics Collaboration (2011). Avoiding bias from weak instruments in Mendelian randomization studies. Int. J. Epidemiol..

