# Supplementary material for: Potential Shared Immunometabolic Signatures Between Familial Hypercholesterolemia and Major Depressive Disorder: Integrative Transcriptomic Analysis, Mendelian Randomization, and Clinical Validation
Source: Int J Mol Sci. 2026 Aug 22;27(17):7532. doi: 10.3390/ijms27177532 (PMC13565513; doi:10.3390/ijms27177532)
Supplement: Supplementary file 1 [file ijms-27-07532-s001.zip › ijms-4395890-supplementary.pdf]

Supplementary material

Table S1. Differentially expressed genes identified in GSE6054, GSE13985, and GSE98793, including P values and Benjamini–Hochberg FDR-adjusted P values.

Dataset	FDR < 0.05 DEGs	Upregulated	Downregulated
GSE6054	200	168	32
GSE13985	0	0	0
GSE98793	6	5	1

Table S2. Summary results of CD4, MRPS21 and CRTC2 in the database

Gene	logFC_GSE6054	P_GSE6054	FDR_GSE6054	logFC_GSE13985	P_GSE13985	FDR_GSE13985	Direction_concordant
CD4	0.456635648	2.75528e-06	0.004628876	0.274941908	0.004211717	0.200342631	TRUE
MRPS21	0.295022104	0.004476156	0.143627808	-0.440175411	0.02162197	0.331086416	FALSE
CRTC2	0.49652012	0.00475897	0.147533494	0.304857636	0.034879945	0.3901097	TRUE

Tabe S3.Meta-analysis results of CD4, MRPS21 and CRTC2

Gene	Meta_logFC	Meta_SE	Meta_CI_lower	Meta_CI_upper	Meta_P	Meta_FDR
CD4	0.370847994135151	0.0535283160440339	0.265932494688845	0.475763493581458	4.26616505449358e-12	1.66380437125249e-10
MRPS21	0.119023140325376	0.0814959919482813	-0.040709004	0.278755284544008	0.144158624876022	0.197964308808622
CRTC2	0.38049530636289	0.0996132354096832	0.185253364959911	0.575737247765869	0.000133599776879468	0.00027137454678642

Table S4. RT-qPCR validation of candidate biomarkers in the four-group clinical cohort.

Gene	Model	FH-MDD vs. MDD Fold Ratio	95% CI low	95% CI high	FH-MDD vs. MDD P
CD4	Unadjusted	2.086	1.275	3.415	0.004
CD4	Non-metabolic covariates	2.051	1.243	3.384	0.005
CD4	Non-metabolic + HAMD	2.046	1.236	3.386	0.006
CD4	BMI + non-metabolic	1.806	1.084	3.010	0.024
CD4	BMI + non-metabolic + HAMD	1.788	1.069	2.991	0.027
CD4	BMI + LDL-C + non-metabolic + HAMD	3.878	0.790	19.032	0.094
MRPS21	Unadjusted	0.733	0.508	1.057	0.096
MRPS21	Non-metabolic covariates	0.715	0.490	1.044	0.082
MRPS21	Non-metabolic + HAMD	0.700	0.482	1.017	0.061
MRPS21	BMI + non-metabolic	0.758	0.514	1.120	0.162
MRPS21	BMI + non-metabolic + HAMD	0.734	0.499	1.081	0.117
MRPS21	BMI + LDL-C + non-metabolic + HAMD	1.073	0.323	3.560	0.908
CRTC2	Unadjusted	1.720	0.856	3.457	0.126
CRTC2	Non-metabolic covariates	1.704	0.825	3.521	0.148
CRTC2	Non-metabolic + HAMD	1.732	0.836	3.588	0.138
CRTC2	BMI + non-metabolic	1.885	0.891	3.988	0.096
CRTC2	BMI + non-metabolic + HAMD	1.945	0.916	4.130	0.083

CRTC2	BMI + LDL-C + non-metabolic + HAMD	3.294	0.318	34.067	0.314

Table S5. Primer sequences and reagents used for RT-qPCR validation:.

(1)Sequences of PCR primers.

Name	Sequence
CD4(human)-F	TTTTCATTGGGCTAGGCATC
CD4(human)-R	ACTGGCAGGTCTTCTTCTCAC
MRPS21-F	CTGTTTTGAAAGCCGATTATTT
MRPS21-R	ACTAGAAAATAAGCAATTTATTTAATT
CRTC2(human)-F	AGGTGATGATGGACATCGGCTCCA
CRTC2(human)-R	GTGGTACTGTTGCCCCCACT

(2)RNA reverse transcription kit:.

PrimeScript™ RT Master Mix RR036A.

(3)Dye-based qPCR reagents:.

Table 820. A.

(4)PBMC Isolation Reagents:.

Figure 180438. Manufacturer: Procell System.

Human peripheral blood lymphocyte culture supernatant: C0025-200ml; Manufacturer: Beyotime.

Table S6. Antibodies for Flow Cytometry Analysis

Name	Catalogue	Manufacturer	Host/Subtype	Excitation/Emission Peak
FcZero-rAb® APC Anti-Human CD4 Rabbit Recombinant Antibody	APC-FcA98042	Proteintech	Rabbit / IgG	650 nm / 660 nm
FcZero-rAb® PE Anti-Human CD3 (OKT3) Rabbit Recombinant Antibody	PE-FcA65569	Proteintech	Rabbit / IgG	496 nm, 565 nm / 578 nm
PerCP-Cyanine5.5 Anti-Human CD45RA (HI100) Monoclonal Antibody	CPY5-65108	Proteintech	Mouse / IgG2b, κ	482 nm / 690 nm
Brilliant Violet 421™ Anti-Human CD197 (CCR7) (G043H7) Monoclonal Antibody	353208	BioLegend	Mouse / IgG2a, κ	405 nm / 421 nm
FcZero-rAb® CoraLite® Plus 488 Anti-Human HLA-DR (L243) Rabbit Recombinant Antibody	CL488-FcA65560	Proteintech	Rabbit / IgG	493 nm / 522 nm

Table S7. Antibodies and instrument information used for flow-cytometric analysis.

Item	Information
Instrument	SA3800 Spectral Cell Analyzer
Manufacturer	Sony Biotechnology
Laser configuration	405, 488, 561, and 638 nm
Fluorochromes analyzed	PE and APC
Acquisition software	SA3800 software
Analysis software	CytExpert version 2.4

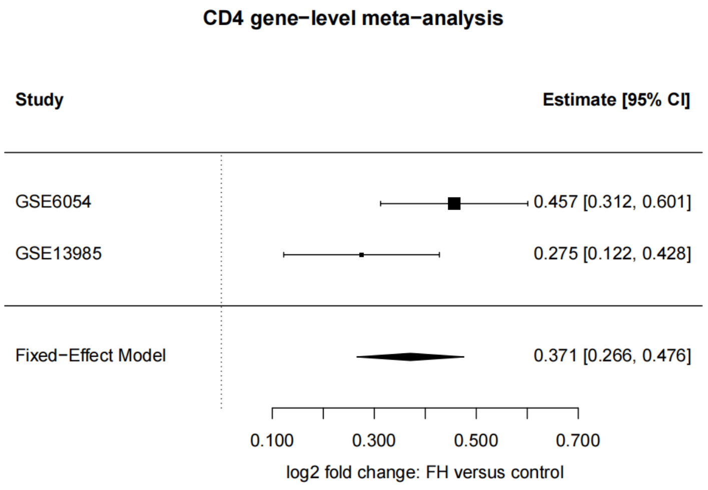

Figure S1. Forest plot for the meta-analysis of CD4.

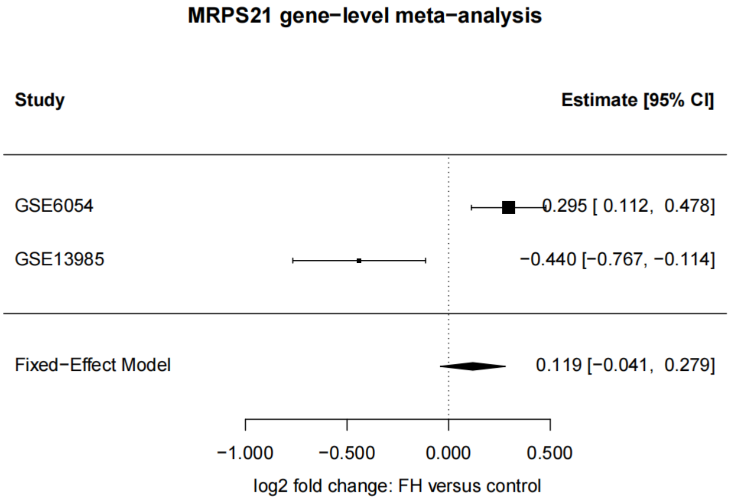

Figure S2. Forest plot for the meta-analysis of MRPS21.

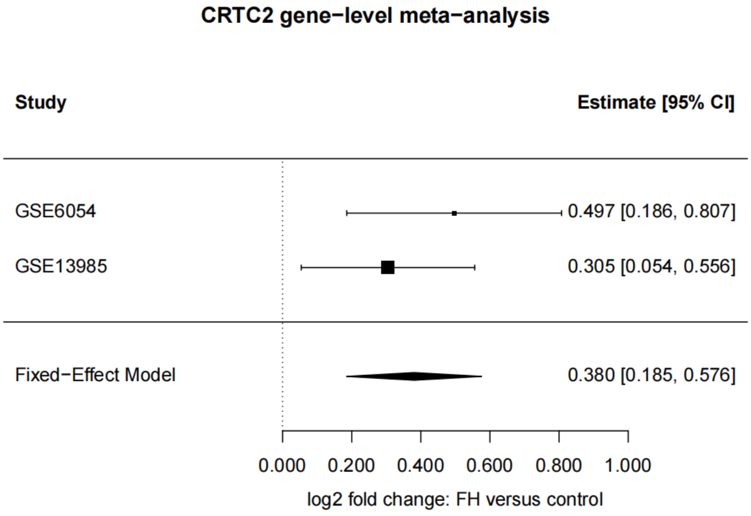

Figure S3. Forest plot for the meta-analysis of CRTC2.
